# Evolution of Minimal Specificity and Promiscuity in Steroid Hormone Receptors

**DOI:** 10.1371/journal.pgen.1003072

**Published:** 2012-11-15

**Authors:** Geeta N. Eick, Jennifer K. Colucci, Michael J. Harms, Eric A. Ortlund, Joseph W. Thornton

**Affiliations:** 1 Institute of Ecology and Evolution, University of Oregon, Eugene, Oregon, United States of America; 2 Howard Hughes Medical Institute, Eugene, Oregon, United States of America; 3 Biochemistry Department, Emory University School of Medicine, Atlanta, Georgia, United States of America; 4 Department of Human Genetics and Department of Ecology and Evolution, University of Chicago, Chicago, Illinois, United States of America; University of Michigan, United States of America

## Abstract

Most proteins are regulated by physical interactions with other molecules; some are highly specific, but others interact with many partners. Despite much speculation, we know little about how and why specificity/promiscuity evolves in natural proteins. It is widely assumed that specific proteins evolved from more promiscuous ancient forms and that most proteins' specificity has been tuned to an optimal state by selection. Here we use ancestral protein reconstruction to trace the evolutionary history of ligand recognition in the steroid hormone receptors (SRs), a family of hormone-regulated animal transcription factors. We resurrected the deepest ancestral proteins in the SR family and characterized the structure-activity relationships by which they distinguished among ligands. We found that that the most ancient split in SR evolution involved a discrete switch from an ancient receptor for aromatized estrogens—including xenobiotics—to a derived receptor that recognized non-aromatized progestagens and corticosteroids. The family's history, viewed in relation to the evolution of their ligands, suggests that SRs evolved according to a principle of minimal specificity: at each point in time, receptors evolved ligand recognition criteria that were just specific enough to parse the set of endogenous substances to which they were exposed. By studying the atomic structures of resurrected SR proteins, we found that their promiscuity evolved because the ancestral binding cavity was larger than the primary ligand and contained excess hydrogen bonding capacity, allowing adventitious recognition of larger molecules with additional functional groups. Our findings provide an historical explanation for the sensitivity of modern SRs to natural and synthetic ligands—including endocrine-disrupting drugs and pollutants—and show that knowledge of history can contribute to ligand prediction. They suggest that SR promiscuity may reflect the limited power of selection within real biological systems to discriminate between perfect and “good enough.”

## Author Summary

The functions of most proteins are defined by their interactions with other biological substances, such as DNA, nutrients, hormones, or other proteins. Some proteins are highly specific, but others are more promiscuous and can interact with a variety of natural substances, as well as drugs and pollutants. Understanding molecular interactions is a key goal in pharmacology and toxicology, but there are few general principles to help explain or predict protein specificity. Because every biological entity is the result of evolution, understanding a protein's history might help explain why it interacts with the substances to which it is sensitive. In this paper, we used ancestral protein reconstruction to experimentally trace how specificity evolved in an ancient group of proteins, the steroid hormone receptors (SRs), a family of proteins that regulate reproduction and other biological processes in animals. We show that SRs evolved according to a principle of minimal specificity: at each point in time, these proteins evolved to be specific enough to distinguish among the substances to which they were naturally exposed, but not more so. Our findings provide an historical explanation for modern SRs' diverse sensitivities to natural and man-made substances; they show that knowledge of history can contribute to predicting the ligands to which a modern protein will respond and indicate that promiscuity reflects the limited power of natural selection to discriminate between perfect and “good enough.”

## Introduction

Cells, like biological entities at higher levels [1], can be viewed as information processing systems, because they change their state or activity in response to specific internal or external cues. This behavior is mediated by functional interactions among the proteins and other molecules that comprise the system [2]. Some proteins are highly specific [3], [4], but others can be regulated by a broader array of molecular partners, including various endogenous ligands, drugs, and pollutants [5], [6].

There has been much speculation about the evolutionary causes of specificity and promiscuity. It is widely believed that evolution usually proceeds from generalist ancestral proteins to more specific recent forms [5], [7]–[10]. Both narrow and broad specificity are often assumed to be the result of optimization by natural selection; according to this view, the capacity of ancient molecules to interact with many partners allowed species with small protein repertoires to carry out a broad set of biological activities and promoted the future evolvability of new functions, while specialization in more recent proteins provides greater efficiency, finer regulation, or prevention of deleterious interactions (refs. [7], [8], [11]–[14], but see ref. [10]).

These hypotheses are largely untested, because there are few natural protein families for which the historical trajectory of changes in specificity has been carefully dissected, although the proximate mechanisms for promiscuous responses have been studied in some extant and engineered proteins [9], [10], [15]. Further, although promiscuous interactions of proteins with exogenous substances are core issues in pharmacology and toxicology, the lack of strong historical case studies means that there are no general principles that explain why molecules have evolved their present-day ligand-recognition criteria. Without such principles, predicting the ligands to which proteins will be sensitive has proven difficult [5], [16].

**Figure 1:**
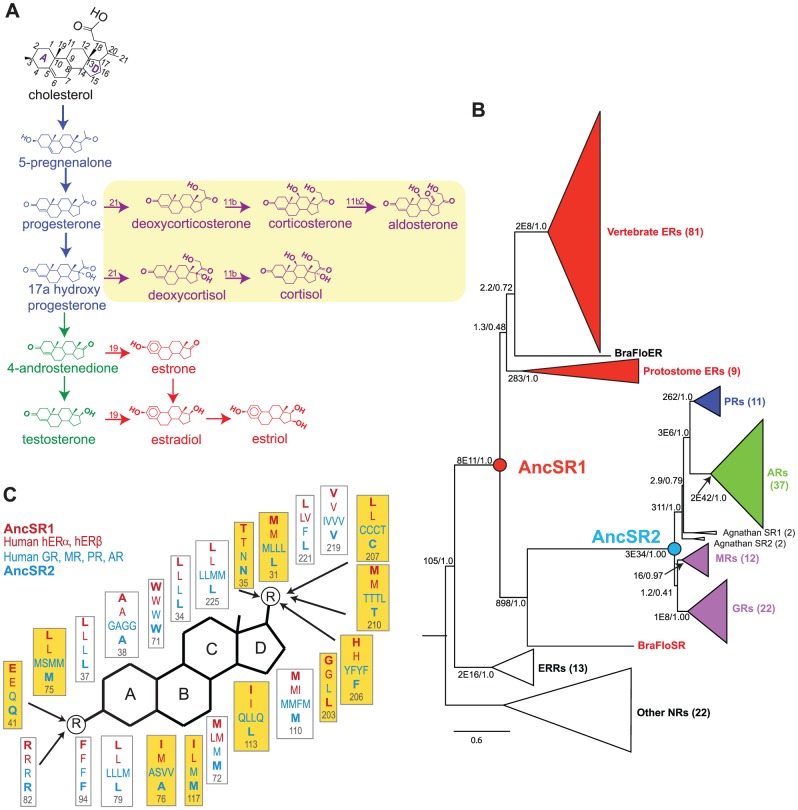
Evolutionary expansion of the steroid receptors and their ligands. A, Pathway for synthesis of vertebrate steroid hormones. The main pathway – synthesis of estrogens (red) via progestagens (blue) and androgens (green) – is at least as ancient as the chordate ancestor. Yellow box, synthesis pathway to corticosteroids (purple), is a later evolutionary novelty found only in vertebrates. The numbering system on the steroid backbone is shown in black. B, Phylogeny of the SR gene family. Receptors are color-coded by the classes of ligands to which they are most sensitive. Ancestral steroid receptors (AncSR1 and AncSR2) resurrected in this study are marked as circles. The number of sequences in each clade is shown in parentheses. Branch supports show approximate likelihood ratios and chi-square confidence metrics for each clade compared to the best phylogeny without that clade. Estrogen-responsive receptors are shown in red. For unreduced phylogenies and a list of sequences, see Figures S10, S11 and Table S7. C, Maximum likelihood reconstruction of ligand-contacting amino acids in AncSR1 and AncSR2, along with residues at homologous sites in extant human SRs. The steroid rings are labeled; circled R indicates polar functional groups at which the major steroid classes differ from each other; arrows indicate residues within hydrogen bonding distance. Residues that differ between AncSR1 and AncSR2 are highlighted in yellow.

Steroid hormone receptors (SRs) are an excellent model for the evolution of specificity. SRs are hormone-activated nuclear transcription factors with distinct specificities for endogenous steroid hormones and exogenous substances. In all SRs, the activating hormone binds in an internal cavity within a well-conserved ligand binding domain (LBD), causing the LBD to change conformation, attract coactivator proteins, and increase transcription of target genes [17]. The SR family diversified through a series of gene duplications that took place during early chordate and vertebrate evolution [18]. Humans have two phylogenetic classes of SRs, which correspond to the chemical classes of endogenous ligands that activate each receptor's LBD. In the first class – the estrogen receptors (ERs) – the endogenous ligands are 18-carbon steroids with an aromatized A-ring and a hydroxyl attached to C3 on the steroid skeleton (Figure 1A). The other class – the nonaromatized steroid receptors (naSRs) – includes receptors for androgens (AR), progestagens (PR), glucocorticoids (GR), and mineralocorticoids (MR); these ligands all contain a nonaromatized A-ring, an additional methyl at C19, and, in most cases, a ketone at C3. Each paralog within the naSR class has distinct specificity based on the size and polarity of the functional groups at C17 and C21 on the steroid's D-ring. Although functional groups at other positions may affect sensitivity, they do not distinguish the classes of ligands recognized by paralogous receptors. SRs also differ in their promiscuous sensitivity to exogenous substances: ERs can be activated by a large set of phenolic drugs and pollutants in diverse chemical classes with highly variant structures, whereas naSRs have far fewer synthetic agonists [19], [20].

Here we characterize in detail the evolutionary trajectory of changes in ligand specificity/promiscuity in the SR protein family, as well as the underlying structural mechanisms for promiscuous responses to non-target ligands. For this purpose, we use ancestral protein resurrection (APR), which uses computational phylogenetic techniques to infer ancestral protein sequences from an alignment of their present-day descendants, followed by gene synthesis, molecular functional assays, and experimental studies of protein structure to directly characterize them. APR represents a powerful strategy for experimentally testing hypotheses about the structure and function of ancient proteins [21], [22]. By dissecting the structure-activity criteria by which ancient receptors distinguished among ligands – and tracing how those criteria changed over time – we sought to gain insight into the evolution of specificity versus promiscuity in the SR family. We also sought to determine whether an understanding of a protein family's history can reveal explanatory principles for understanding and predicting the ligands to which its members will respond.

## Results/Discussion

### Reconstruction and characterization of ancestral proteins

To understand how and why the differences in ligand specificity between the ERs and naSRs receptors evolved, we used ancestral protein resurrection [21] to experimentally characterize the LBDs of two key ancient members of the protein family. AncSR1 is the last common ancestral protein from which the entire SR family descends by a series of gene duplications; AncSR2 is the ancestral protein of all naSRs (Figure 1B). The family's phylogeny indicates that both proteins are hundreds of millions of years old: AncSR1 predates the divergence of vertebrates from other chordates, and AncSR2 predates the divergence of jawed vertebrates from jawless fishes [18].

From alignments of ∼200 extant receptor proteins, we used likelihood-based phylogenetic methods to infer the best-fitting evolutionary model, phylogeny, and ancestral protein sequences. The sequence of AncSR2 was reconstructed with high confidence (mean posterior probability (PP) = 0.93 per site, Figure S1, Table S1), and even less ambiguity at ligand-contacting sites (mean PP = 0.96). AncSR1 was more ambiguously reconstructed (mean PP = 0.70 overall, Figure S2, Table S2), but at ligand-contacting sites its reconstruction was considerably more robust (mean PP = 0.90).

The AncSR1 sequence is most similar to those of the extant ERs, whereas that of AncSR2 is most similar to the naSRs, and this pattern is most pronounced at sites in the ligand-contacting pockets (Figure 1C, Table S3). These findings suggest that AncSR1 may have been activated by estrogens and AncSR2 by nonaromatized steroids, a scenario also supported by the phylogenetic distribution of ligand specificities among extant receptors – particularly the presence of estrogen-sensitive receptors in invertebrates such as annelids and cephalochordates [18], [23].

**Figure 2:**
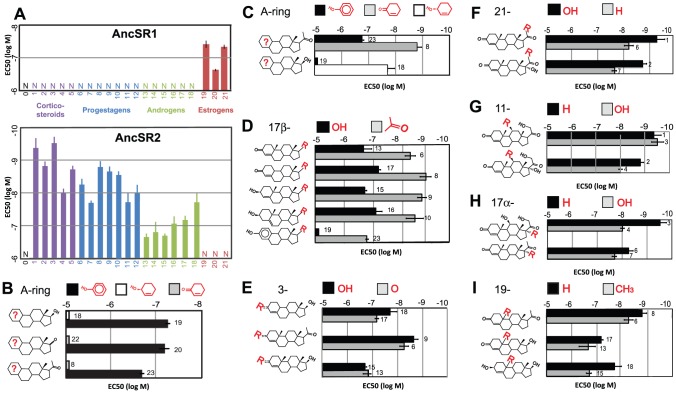
Ligand-recognition rules of AncSR1 and AncSR2. A, The sensitivity of AncSR1-LBD (top panel) and AncSR2-LBD (bottom panel) to various hormones (Table S4) was characterized in a triplicate luciferase reporter assay and is displayed as EC50, the concentration at which half-maximal reporter activation is achieved. Error bars, 95% confidence interval. Sets of hormones are grouped by color and are numerically labeled according to the list below. B, AncSR1's ligand recognition criteria. Each pair of bars shows the EC50 of AncSR1 to a pair of hormones that differ only by aromatization of the A-ring (shown in red on the ligand structure and in the key). Unlike aromatization, substitution of a 17-keto or acetyl for estradiol's hydroxyl has only a weak effect on sensitivity, as shown by the small differences among pairs. C-I, AncSR2's ligand recognition criteria. Each pair of bars shows the sensitivity of the receptor to hormones that differ only in the functional group at specified positions or aromatization of the A-ring. Bar labels indicate the substance tested: 0, cholesterol, 1, 11-deoxycorticosterone, 2, 11-deoxycortisol; 3, corticosterone; 4, cortisol; 5, aldosterone; 6, progesterone; 7, 17α-hydroxyprogesterone; 8, 19-norprogesterone; 9, 4-pregnenolone; 10, 5-pregnenolone; 11, 20α hydroxyprogesterone; 12, 20β hydroxyprogesterone; 13, testosterone; 14, dihydrotestosterone; 15, 4-androstenediol; 16, 5-androstenediol; 17, 19-nortestosterone; 18, bolandiol; 19, estradiol; 20, estrone; 21, estriol; 22, 4-androstenedione; 23, 19-nor-1, 3, 5(10)-pregnatriene-3-ol-20-one (NPT).

To experimentally test these hypotheses, we synthesized cDNAs for the AncSR1 and AncSR2 LBD protein sequences, expressed them as Gal4-DBD fusion constructs, and characterized their sensitivity to hormones using luciferase reporter gene assays. As predicted, we found that AncSR1 is a highly specific estrogen receptor, activating transcription in the presence of nanomolar concentrations of physiological estrogens. It was unresponsive to a broad array of androgens, progestagens, and corticosteroids, as well as cholesterol (Figure 2A, Figure S3). In contrast, AncSR2 was completely unresponsive to estrogens (and cholesterol) but strongly activated by low concentrations of diverse nonaromatized steroid hormones, including progestagens and corticosteroids and – to a lesser extent – androgens (Figure 2A, Figure S4). We also experimentally characterized numerous alternative reconstructions of AncSR1 and AncSR2 and found that these proteins' specificities for aromatized and nonaromatized steroids, respectively, are highly robust to uncertainty in the reconstruction (Figures S5, S6).

We conclude that a fundamental inversion of ligand specificity for endogenous steroid hormones – not a narrowing of specificity from a promiscuous ancestor – took place during the evolutionary interval between AncSR1 and AncSR2. This inversion must have occurred in the lineage leading to vertebrates after they diverged from cephalochordates, because cephalochordates possess a single naSR ortholog, which retains the ancestral specificity for estrogens (Figure 1B, see [24]). Subsequently, the promiscuous responses of AncSR2 to nonaromatized steroids were differentially partitioned among its descendant lineages to yield the more specific PR, GR, MR, and AR. In extant receptors, mutations that make these SRs sensitive to the ligands of other members of the family now now cause deleterious phenotypes [25]–[27].

Our findings, viewed in the context of the ancient pathway for steroid hormone synthesis, suggest that some hormone-receptor pairs were assembled during evolution by a process of molecular exploitation, whereby molecules with a different ancient function are recruited into new signaling partnerships after gene duplication and/or divergence [23], [28]. That the ancient AncSR1 was specific for estrogens implies that progestagens and androgens, which are intermediates in the synthesis of estrogens (Figure 1A), existed before steroid receptors evolved to transduce their signals. When AncSR2 and its descendants evolved the capacity to be activated by nonaromatized steroids, these biochemical steppingstones in estrogen synthesis were recruited into new, bona fide signaling partnerships.

### Ancestral structure-activity criteria

The specificity of a protein can be described by the biochemical criteria by which it distinguishes between functionally relevant binding partners and all other substances. To dissect more precisely how the ligand-recognition criteria of SRs evolved during the interval between AncSR1 and AncSR2, we applied a structure-activity approach. We characterized the specificity of these two ancestral proteins using a library of synthetic and natural steroids that differ from each other only by the aromatization of the A-ring or the functional groups at specific positions that vary among physiological steroids (Figure 2, Table S4).

We found that AncSR1's specificity is determined primarily by a single major criterion: requirement for an aromatized A-ring. All aromatized steroids tested activated AncSR1, but no natural nonaromatized steroids were effective at nanomolar concentrations (Figure 2A, Figure S3). Comparisons using several matched pairs of aromatized/nonaromatized steroids confirm that AncSR1 distinguishes strongly among potential ligands based on its requirement for an aromatized A-ring, with EC50s that increase by orders of magnitude when only this aspect of the ligand is changed (Figure 2B, Table S5). Beyond this major criterion, AncSR1's specificity is rather loose. In particular, it tolerates different functional groups around the D-ring, as shown by its similar sensitivity to estradiol and estrone, which contain a 17-hydroxyl and ketone, respectively (Figure 2A, 2B). Even the “chimeric” steroid 19-nor-1, 3, 5(10)-pregnatriene-3-ol-20-one (NPT) – which has the much larger 17β-acetyl group found on progesterone and corticosteroids – is almost as potent an AncSR1 activator as endogenous estrogens (Figure 2B).

AncSR2's ligand-recognition criteria differ from AncSR1's in two major ways (Figure 2, Table S5). First, AncSR1's A-ring rule is inverted in AncSR2, which is more sensitive to nonaromatized steroids than to otherwise identical aromatized substances by two to three orders of magnitude (Figure 2C). Second, AncSR2 evolved an additional criterion: it prefers steroids with a 17β-acetyl group (such as progestagens and corticosteroids) to those with smaller hydroxyls or ketones (androgens and estrogens), as demonstrated by the 21- to 87-fold difference in EC50 values between pairs of hormones that differ only at this position (Figure 2D).

Beyond these two criteria, AncSR2's specificity is rather loose (Figure 2E–2I). AncSR2 does not distinguish strongly between progestagens and corticosteroids because it has only a weak preference for steroids with a 21-hydroxyl (Figure 2F). The presence/absence of an 11-hydroxyl, present on many corticosteroids, does not strongly affect the receptor's sensitivity (Figure 2G). AncSR2 does not distinguish between 3-hydroxy and 3-ketosteroids, so long as the A-ring is not aromatized (Figure 2E), and it does not require the 19-methyl present on endogenous nonaromatized steroids (Figure 2H). Taken together, these data indicate that the evolution of AncSR2's ligand specificity entailed two major changes: inversion of AncSR1's fundamental ligand-recognition criterion for an aromatized A-ring and acquisition of an additional criterion at the D-ring.

### Minimal specificity in SR evolution

The evolving ligand recognition rules of AncSR1 and AncSR2 can be understood in light of existing knowledge concerning the biosynthesis and evolution of the ligands. Taken together, our findings suggest that the evolution of the SR family has been characterized by minimal specificity, a concept borrowed by analogy from information theory [29]: each receptor evolved to be specific enough to distinguish among the set of contemporaneous endogenous ligands to which it was exposed, but not more so.

**Figure 3:**
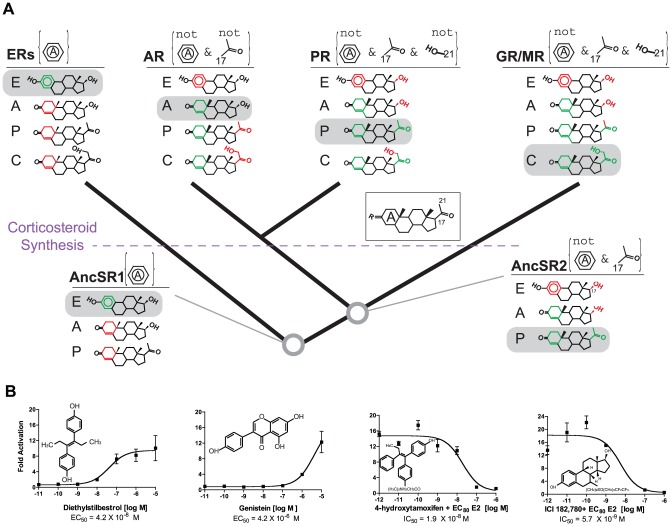
Evolution of minimal specificity. A, Evolution of ligand-recognition criteria on the SR phylogeny. For each ancient and extant receptor, the criteria that distinguish activating ligands from other endogenous steroids are shown in brackets. Rules labeled “not” indicate significantly strongly reduced sensitivity when the specified moiety is present; other rules indicate strongly increased sensitivity when the moiety is present. The structures of representative endogenous hormones – estrogens (E), androgens (A), progestagens (P) and corticosteroids (C) – that were synthesized at each point in time are shown. Green portions of each hormone show moieties that satisfy the receptor's rules; red portions violate rules. Each receptor's rules are sufficient to allow activation by only a single class of hormones (gray boxes). The evolution of corticosteroid synthesis is indicated; AncSR2's criteria would not have been sufficient to distinguish corticosteroids from progestagens. Inset: common steroid structure with A-ring and key carbons labeled. Dose-response curves for extant receptors are shown in Figure S7. B, AncSR1 is activated/antagonized by xenoestrogens in a luciferase reporter assay. IC50, concentration at which half-maximal inhibition was achieved in the presence of estradiol (EC_80_ = 200 nM). Each point shows the mean and SEM of three replicates.

The concept of minimal specificity provides an evolutionary explanation for the specificity and promiscuity possessed by each receptor. For example, AncSR1's single criterion – requiring an aromatized A-ring – provided minimally sufficient specificity for estrogens (Figure 3A). Estrogens are the only aromatized steroids produced in animals, because aromatization of the steroid A-ring is the final step in a conserved estrogen synthesis pathway beginning with cholesterol and proceeding via progestagens and androgens as intermediates (Figure 1A). AncSR1's simple criterion therefore allowed it to exclude all other endogenous steroids, including androgens, progestagens, and cholesterol and its metabolites. These hormones are all ancient: synthesis of estrogens via progestagens and androgens is as old as the ancestor of cephalochordates and vertebrates [30], and it may be even older, given the presence of all these hormones in mollusks [31].

Minimal specificity is also apparent in the evolution of AncSR2 and its descendants (Figure 3A, Figure S7). When AncSR2 became sensitive to nonaromatized steroids, it would have excluded estrogens but become sensitive to both progestagens and androgens; acquiring its second ligand-recognition rule restricted AncSR2's sensitivity to progestagens only. AncSR2 did not yet distinguish progestagens from corticosteroids, but endogenous synthesis of these steroids had not yet evolved; only later – during or after the same period of early vertebrate evolution when synthesis of corticosteroids first evolved due to the emergence of 21-hydroxylase activities in the CYP450 family [30], [32] – were AncSR2's promiscuous sensitivities partitioned among the PR, GR, and MR.

These data indicate that each receptor evolved ligand recognition criteria sufficiently complex to parse the repertoire of ligands present during its evolution, but those rules were not sufficient to prevent promiscuous responses to other substances that had not yet evolved. By evolving narrower specificity as the synthesis of new steroids emerged during vertebrate evolution, the various SRs presumably maintained the capacity to transduce specific signals despite the organism's increasing chemical repertoire (Figure 3A).

### An evolutionary explanation for SR-mediated endocrine disruption

Predicting ligands that interact with intended and secondary protein targets is an important goal in pharmacology and toxicology, but understanding from first principles which targets will respond more or less promiscuously has proven difficult [5], [16]. The concept of minimal specificity predicts that ER's capacity to be disrupted by exogenous phenolics is inherited from AncSR1. To test this possibility, we characterized the ability of several xenoestrogens to activate AncSR1. As predicted, we found that AncSR1 is activated by the strong nonsteroidal ER agonists diethylstilbestrol and genistein and is competitively inhibited by the ER antagonists 4-hydroxytamoxifen and ICI182780 (Figure 3B).

Our observations provide an historical explanation for the greater susceptibility of ERs than naSRs to activation by pollutants, pharmaceuticals, and dietary compounds. Extant ERs inherited AncSR1's simple ligand-recognition criterion requiring little more than an aromatized A-ring with a 3-hydroxyl (Figure S8). Although this rule provided sufficient specificity throughout virtually all of vertebrate evolution, ERs are now exposed to – and fortuitously activated by – a wide range of aromatized pharmaceutical, industrial, and agricultural substances of the appropriate size and shape that have come into large-scale production only in the last century [19].

In contrast, the more restrictive specificity of AR, PR, GR, and MR – which reflects the greater variety of endogenous potential activators to which they were exposed during evolution – makes them susceptible to activation by fewer synthetic substances than ERs, although they can still be disrupted by some novel substances, such as nonaromatized 19-norsteroids used as synthetic androgens. As predicted, we found that AncSR2, like its descendants, is insensitive to the aromatized xenoestrogens (Figure S9).

Taken together, our findings suggest that analysis of a protein's history and the chemical milieu in which it evolved can provide useful information for predicting the endogenous and exogenous ligands that can interact with it.

### Structural causes of SR promiscuity

**Figure 4:**
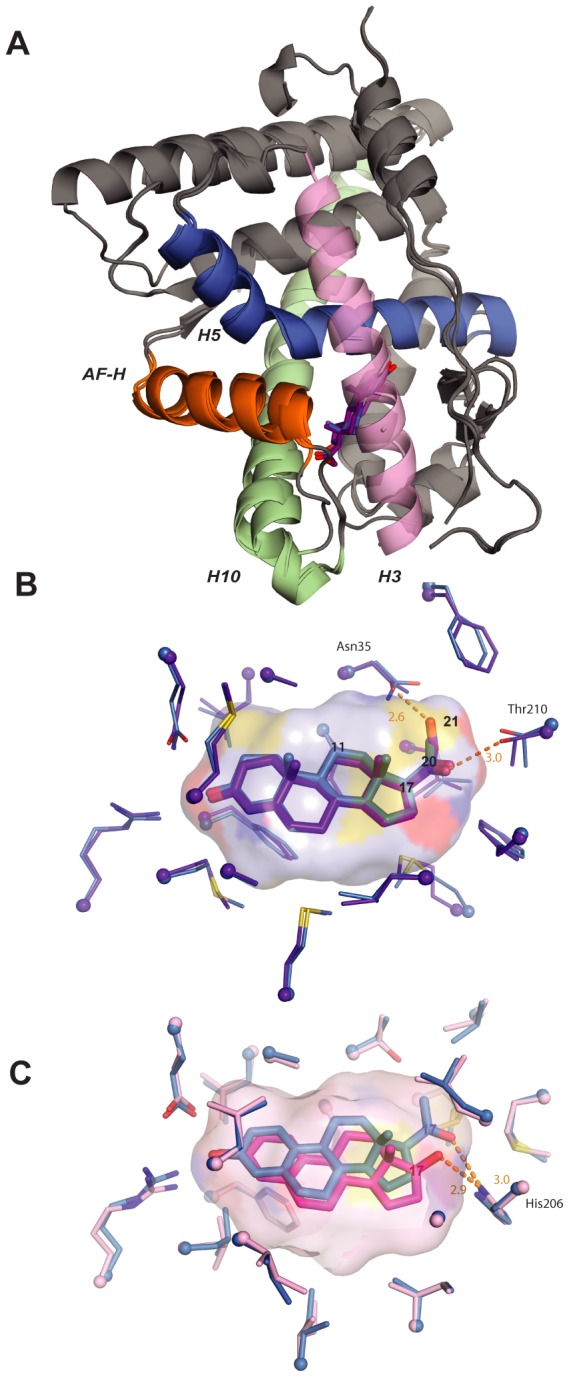
Structural causes of minimal specificity. A, X-ray crystal structures of AncSR2 with progesterone (blue) and DOC (purple) are superimposed. Ligands are shown as sticks. Helices making major ligand contacts and the activation-function helix (AF-H) are shown in contrasting colors. B, Structural causes of promiscuity in AncSR2. The ligand cavity of the AncSR2-progesterone structure, shown as a surface, has adequate volume to accommodate the 21-hydroxyl of DOC. Ligand contacts in the crystal structures of AncSR2 with progesterone (blue) and DOC (purple) are shown. Thick sticks, ligand; thin sticks, side chains that contact ligand; balls, α-carbons. Steroid carbons 11, 17, 20, and 21 are numbered. Hydrogen bonds are shown as orange dotted lines. C, Structural basis for promiscuity in AncSR1. Ligand contacts in the AncSR1 model with estradiol (magenta) and NPT (blue) are shown. The cavity of the AncSR1-estradiol complex, which has adequate room to accommodate the 17-acetyl of NPT, is shown. Two side chains between the viewer and the ligand are hidden for clarity.

Finally, we sought to understand the underlying features of protein structure that caused AncSR1's and AncSR2's promiscuous responses associated with minimal specificity. We first used X-ray crystallography to determine the structures of bacterially expressed AncSR2-LBD in complex with progesterone and with 11-deoxycorticosterone (DOC), at 2.75 and 2.82 Å resolution, respectively (Figure 4A, Table S6). The structures reveal why AncSR2 did not yet distinguish between progestagens and corticosteroids, which differ only in that the latter contain a 21-hydroxyl. The two protein backbones have nearly identical topologies (RMSD = 0.28 Å), and there are virtually no differences in the ways the ligands are bound (Figure 4A, Table S6). The AncSR2-progesterone complex contains ample room to accommodate the additional 21-hydroxyl of corticosteroids (Figure 4B). Further, Asn35 offers a perfectly positioned hydrogen bond partner, which is unpaired in the AncSR2-progesterone complex, for DOC's hydroxyl (Figure 4B). This additional favorable interaction explains why AncSR2 not only accommodates corticosteroids but is even more sensitive to them than progestagens.

To understand the structural causes of AncSR1's inability to distinguish between 17-hydroxyl and 17-acetyl steroids, we used homology modeling/energy minimization based on a human ERα template to predict the AncSR1-LBD structure in complex with estradiol and NPT. Despite differing by 172 amino acids, AncSR1 and AncSR2 have remarkably similar peptide backbone conformations (RMSD = 0.87 Å). AncSR1's capacity to adventitiously accommodate larger 17-acetyl steroids appears to be due to excess volume and hydrogen bonding capacity in AncSR1's cavity near the ligand's D-ring. When NPT is docked in the AncSR1 cavity, virtually no adjustment is required in the position of nearby residues compared to those in the estradiol complex: instead, the long axis of the ligand moves slightly towards H10, allowing NPT's larger acetyl group to slot into space that was unoccupied in the estradiol complex (Figure 4C). Further, the 20-keto of NPT accepts a hydrogen bond from His206, which can serve as a donor (as in the NPT complex), acceptor (as in the estradiol complex), or both, depending on its ionization state.

Taken together, these data indicate that the promiscuous responses of both AncSR1 and AncSR2 to non-target ligands are due in large part to unfilled volume in the internal cavity and untapped potential of polar side chains to form hydrogen bonds with polar atoms on the ligand [5], [9].

### Promiscuity, selection, and neutrality in the evolution of signaling

The promiscuity we observed during SR evolution appears to reflect the fact that there is no functional difference between a receptor that excludes ligands to which the cell is never exposed and a more promiscuous receptor that does not possess such ligand recognition criteria. Although ancient and extant SRs are only minimally specific, their potential promiscuity would not have caused them to transduce noisy signals in their historical chemical environments, because such signals were not rampantly produced at the time; there would presumably have been be no fitness cost or benefit associated with the specific forms of promiscuity these receptors manifested. Rather than representing an optimum, then, the imperfect specificity of each SR appears to reflect the limited power of selection to distinguish between “perfect” and “good enough,” given the chemical context in which these proteins evolved. Our findings are related to prior work suggesting that other protein properties, such as marginal stability, may not be uniquely adaptive states but may instead reflect the limited power of selection to optimize a property that affects fitness only when the property is near a threshold [33].

We predict that minimal specificity will be apparent in many other protein families. Protein engineering studies have shown that enzymes in the laboratory often neutrally evolve promiscuous responses to substrates not yet present in the system [9], [15]. Further, the limited specificity of natural proteins is what allows them to respond to novel drugs and xenobiotic pollutants. Direct study of historical evolution in other protein families and their ligands is necessary to determine the generality of the principle of minimal specificity and to characterize the dynamics that have shaped proteins' natural specificity and their responses to drugs and pollutants.

A phenomenon similar to minimal specificity is well known in biological information systems at higher levels, such as choice by individuals of conspecific mates [34] and mimics that lure prey or pollinators by exploiting a receiving species' signal recognition capacity [35], [36]. In each case, the “receptor” distinguishes target from nontarget signals in the species' environment but fails to exclude novel signals to which it has not previously been exposed. Minimal specificity, reflecting evolution in the face of the limited set of stimuli present in real environments, may therefore be a general characteristic of signaling and information systems from molecular to community scales.

## Methods

### Phylogenetics and ancestral sequence reconstruction

Annotated protein sequences for nuclear receptors were downloaded from UniPROTKB/TrEMBL, GenBank, the JGI genome browser, and Ensemble (Table S7). For the reconstruction of AncSR2, 184 steroid and related receptor sequences containing both DNA binding and ligand binding domains were aligned using the Multiple Sequence Alignment by Log-Expectation (MUSCLE) program [37]. The alignment was checked to ensure alignment of the nuclear receptor AF-2 domain and manually edited to remove lineage-specific indels. The N-terminal variable region and hinge region were removed from the alignment file, as these areas could not be aligned reliably among sequences. AncSR1 was reconstructed using an expanded alignment (213 sequences), reflecting the deposition of many new SR sequences in public databases since a much earlier study of AncSR1 [23].

Phylogenies (Figures S10, S11) were inferred from these alignments using PHYML v2.4.5 [38] and the Jones-Taylor-Thornton model with gamma-distributed among-site rate variation and empirical state frequencies, which was the best-fit evolutionary model selected using the Akaike Information Criterion implemented in PROTTEST software. Statistical support for each node was evaluated by obtaining the approximate likelihood ratio (the likelihood of the best tree with the node divided by the likelihood of the best tree without the node) and the chi-squared confidence statistic derived from that ratio [39].

AncSR1 and AncSR2 were initially reconstructed by the maximum likelihood method [40] on the ML phylogeny for each alignment using the Codeml module of PAML v3.14 [41] and Lazarus software [42], assuming a free eight-category gamma distribution of among-site rate variation and the Jones-Taylor-Thornton protein model. AncSR2 was also reconstructed on a single-branch rearrangement of the ML phylogeny that requires fewer gene duplications and losses to explain the distribution of SRs in agnathans and jawed vertebrates (Figure S12, Table S8). Average probabilities were calculated across all LBD sites except those containing indels.

### Reporter activation assays

cDNAs coding for the maximum likelihood AncSR2 LBD and AncSR1 LBD were synthesized (Genscript) and verified. The LBDs were then cloned into the Gal4-DBD-pSG5 vector; 31 amino acids of the GR hinge containing the nuclear localization signal-1 [43] were inserted between the DBD and LBD to ensure nuclear localization and conformational independence of the two domains. The hinge and ligand-binding domain (LBD) of the human progesterone receptor (hPR; aa 632–933; Swiss-Prot P06401), human estrogen receptor alpha (hERα, aa 435–595; Swiss-Prot P03372, [44]), human glucocorticoid receptor (hGR; aa 485–777; Swiss-Prot P04150, [45]), human mineralocorticoid receptor (hMR, aa 736–984, Swiss-Prot P08235; [45]) were cloned into the Gal4-DBD-pSG5 vector in frame with the Gal4 DBD. The human androgen receptor (hAR) LBD was cloned into the pFN26A (BIND) hRluc-neo Flexi Vector (Promega) without the hinge domain (aa 671–919; Swiss-Prot P10275), as the hinge domain of the hAR inhibits AF-2 dependent activation of the hAR [46].

The hormone-dependent transcriptional activity of resurrected ancestral receptors and their variants as well as the human receptor LBDs was assayed using a luciferase reporter system. CHO-K1 cells were grown in 96-well plates and transfected with 1 ng of receptor plasmid, 100 ng of a UAS-driven firefly luciferase reporter (pFRluc), and 0.1 ng of the constitutive pRLtk *Renilla* luciferase reporter plasmid, using Lipofectamine and Plus Reagent in OPTIMEM (Invitrogen). After 4 h, transfection medium was replaced with phenol-red-free αMEM supplemented with 10% dextran-charcoal stripped FBS (Hyclone). After overnight recovery, cells were incubated in triplicate with the hormone of interest from 10∧−12 to 10∧−5 M for 24 h, then assayed using Dual-Glo luciferase (Promega). Firefly luciferase activity was normalized by *Renilla* luciferase activity. Dose-response relationships were estimated using nonlinear regression in Prism4 software (GraphPad Software, Inc.); fold increases in activation were calculated relative to the vehicle-only (ethanol) control.

### Alternative ancestral reconstructions

To determine the robustness of functional inferences to statistical uncertainty in the reconstruction of AncSR1 and AncSR2, we used two approaches. AncSR1 had too many ambiguously reconstructed sites to examine each such residue individually, so we computationally sampled from the posterior probability distribution of reconstructed amino acid states to generate a cloud of possible ancestral sequences, each harboring a large number of alternate states. Specifically, we generated 1,000,000 possible ancestral sequences by sampling from the posterior probability distribution of states at each site. Of this sample, the five sequences with the highest total posterior probability differed from the ML reconstruction at 55 to 59 sites and from each other by 63 to 82 sites; these sequences had total posterior probabilities lower than AncSR1-ML by a factor of 10^−23^ to 10^−24^. They differed from each other at several sites in the ligand pocket and included four unique combinations of ligand-contacting residues. We synthesized these five radically alternative ancestral reconstructions de novo and repeated the functional assays. Despite their extreme distance from AncSR1-ML, all five alternative reconstructions were sensitive to estrogens and did not respond to nonaromatized steroids (Figure S5).

For AncSR2, we identified all plausible alternate reconstructions (those with posterior probability >0.20 excluding biochemically similar K/R, D/E, S/T, and I/L differences) and introduced each alternate state individually into the AncSR2 background using the Quikchange Mutagenesis kit (Stratagene), verified clones by sequencing, and repeated the activation assays with each version of AncSR2 (Figure S6). The ML AncSR2 sequence reconstructed on the ML tree had high baseline activation in the absence of ligand; this phenotype is almost certainly an artifact, because constitutive baseline activity is not present in any of AncSR2's extant descendants; it is well-established that some amino acid replacements can cause nuclear receptors to become constitutive by marginally stabilizing the active conformation in the absence of hormone [47]. We therefore introduced all plausible alternate reconstructions into AncSR2-ML and found that one (L79M) eliminated this ligand-independent activity. The “constitutive” Leu79 state is weakly supported on the ML tree (PP = 0.59), and has no support (PP = 0.00) on the phylogeny that is most parsimonious in terms of gene duplications and losses; in contrast, the “non-constitutive” state Met79 has PP = 0.41 on the ML tree and PP = 1.00 on the rearranged gene duplication/loss tree (Figure S12, Table S8). The AncSR2 sequence used for all experiments reported in the text therefore contains state Met79. The other alternate reconstructions were then reintroduced into this AncSR2 sequence: none qualitatively changed the receptor's sensitivity to the various classes of steroid hormones, except for A171V, which conferred constitutive activity (Figure S6).

### Protein expression

The AncSR2 ligand binding domain (LBD) cDNA (residues 1–252) was cloned into pLIC-MBP (provided by J. Sondek, Chapel Hill, NC), which contains a hexahistadine tag followed by the maltose binding protein (MBP) and a tobacco etch virus (TEV) protease site N-terminal to the protein. AncSR2 was expressed as a fusion protein in BL21(DE3) pLys cells in the presence of 50 µM ligand using standard methods, and initially purified using affinity chromatography (HisTrap columns, GE Healthcare). Following TEV cleavage, the tagged MBP was removed by an additional nickel affinity column. AncSR2 was purified to homogeneity via gel filtration. Pure AncSR2 LBD was dialyzed against 150 mM sodium chloride, 20 mM Tris HCl (pH 7.4), 5% glycerol, and 50 µM CHAPS and concentrated to 2–5 mg/mL.

### Crystallization and structural analysis

Crystals of AncSR2-LBD with ligand were grown by hanging drop vapor diffusion at 22°C from solutions containing 1.0 µL of protein at 2–5 mg/mL protein and 1.0 µL of the following crystallant: 0.8–1.2 M MgSO_4_, 6–12% glycerol, and 100 mM MES, pH 5.4–6.4. Orthorhombic crystals of the AncSR2 – progesterone and 11-DOC complex grew in P2_1_2_1_2_1_ and C222_1_ spacegroups with either two monomers or one monomer in the asymmetric unit, respectively.

Crystals were cryoprotected in crystallant containing 20% glycerol and were flash-cooled in liquid N_2_. Data to 2.75 Å and 2.82 Å resolution were collected for the AncSR2-progesterone and AncSR2-deoxycorticosterone complexes, respectively (Table S6). All data were collected at South East Regional Collaborative Access Team (SER-CAT) 22-ID at the Advanced Photon Source at Argonne National Laboratory in Chicago, IL, and were processed and scaled with HKL2000 (HKL Inc.). Initial phases for the AncSR2- progesterone complex were determined using a homology model to the progesterone receptor (1A28) as the initial search model in Phenix (Phenix) [48]. Subsequent structures were solved using the best available AncSR2 structure for initial phases. All structures were refined using standard methods in the CCP4 suite of programs and COOT v0.9 was used for model building [49]. Omit maps were generated by removing coordinates corresponding to the ligand and running 10 rounds of restrained refinement in CCP4. Maps are contoured to 1 σ (Figure S13). Figures were generated using PyMol (Schrödinger, LLC). AncSR2 structures with progesterone and DOC have PDB accessions 4FN9 and 4FNE, respectively. Structures were rendered for display using Pymol software.

The structure of AncSR1-LBD was predicted by homology modeling, based on a human ERα∶estradiol structure (1ERE), the most similar human receptor in sequence and function. We used Modeller software [50] to infer the AncSR1-LBD structure 100 times, chose the lowest-energy iteration from these structures, and verified it using RAMPAGE software [51], which showed only 4/237 Ramachandran outliers, all of which were in surface loops. Cavity volumes were inferred using VOIDOO software [52] by calculating the volume accessible to a probe 1.4 Å in diameter.

## Supporting Information

Figure S1 Histogram of distribution of posterior probabilities for AncSR2 and posterior probabilities of amino acid residues lining the binding pocket.
(PDF)

Figure S2 Histogram of distribution of posterior probabilities for AncSR1 and posterior probabilities of amino acid residues lining the binding pocket.
(PDF)

Figure S3 Representative dose activation curves of AncSR1 in response to cholesterol and a library of hormones (#0-23).
(PDF)

Figure S4 Representative dose activation curves of AncSR2 in response to cholesterol and a library of hormones (#0-23).
(PDF)

Figure S5 The specificity of AncSR1 is robust to uncertainty in the reconstruction.
(PDF)

Figure S6 The specificity of AncSR2 is robust to uncertainty in the reconstruction.
(PDF)

Figure S7 Sensitivities of extant human receptors to an estrogen, androgen, progestagen, and corticosteroid.
(PDF)

Figure S8 Activation of the estrogen receptor ligand binding domains of two annelids and human ERα.
(PDF)

Figure S9 AncSR2 is not activated by the nonsteroidal ER agonists diethylstilbestrol and genistein and is not inhibited by ICI182870 and 4-hydroxytamoxifen.
(PDF)

Figure S10 Unreduced ML steroid receptor phylogeny based on alignment of 184 steroid receptors and related sequences used to reconstruct AncSR2.
(PDF)

Figure S11 Unreduced ML steroid receptor phylogeny based on alignment of 213 steroid receptors and related sequences used to reconstruct AncSR1.
(PDF)

Figure S12 Unreduced 184-taxon steroid receptor gene duplication phylogeny.
(PDF)

Figure S13 Omit maps showing that progesterone and 11-deoxycorticosterone bind directly to AncSR2 to promote receptor activation.
(PDF)

Table S1 The reconstructed sequence of AncSR2.
(PDF)

Table S2 The reconstructed sequence of AncSR1.
(PDF)

Table S3 Percent similarity of the ligand-binding domains of AncSR1 and AncSR2 to those of extant steroid receptors in humans.
(PDF)

Table S4 Pubmed compound identifier (CID) numbers for cholesterol and the synthetic and natural steroid hormones tested in this study.
(PDF)

Table S5 Fold preferences of AncSR1 and AncSR2 for the hormone pairs indicated in Figure 2.
(PDF)

Table S6 Data collection and refinement statistics for the AncSR2 crystal structure in complex with 11-DOC and progesterone.
(PDF)

Table S7 List of receptors and the organisms they were isolated from used in this study.
(PDF)

Table S8 Comparison of the sequence of AncSR2 as reconstructed on the ML phylogeny and gene duplication phylogeny.
(PDF)
