# Supplementary material for: Evolution of Minimal Specificity and Promiscuity in Steroid Hormone Receptors
Source: PLoS Genet. 2012 Nov 15;8(11):e1003072. doi: 10.1371/journal.pgen.1003072 (PMC3499368; doi:10.1371/journal.pgen.1003072)
Supplement: Figure S1 — Histogram of distribution of posterior probabilities for AncSR2 and posterior probabilities of amino acid residues lining the binding pocket. (PDF) [file pgen.1003072.s001.pdf]

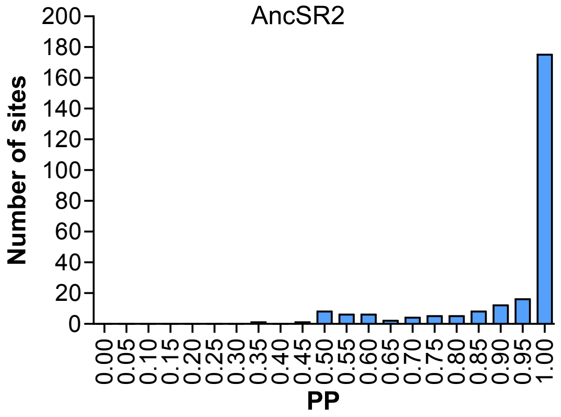

AncSR2 Binding Pocket

Position	Reconstructed Amino Acid	Probability	Alt State #1	Probability
41	**Q**	1.00		
75	**M**	1.00		
37	**L**	1.00		
38	**A**	0.87	C	0.06
71	**W**	1.00		
34	**L**	1.00		
225	**L**	0.77	M	0.23
35	**N**	1.00		
31	**L**	1.00		
221	**F**	1.00		
219	**V**	1.00		
207	**C**	1.00		
210	**T**	1.00		
206	**F**	1.00		
203	**L**	1.00		
110	**M**	1.00		
113	**L**	1.00		
72	**M**	1.00		
117	**M**	1.00		
76	**A**	0.88	S	0.05
79	**L**	0.59	M	0.40
94	**F**	1.00		
82	**R**	1.00		
	**Mean Posterior Probability**	**0.96**		

Fig. S1 Histogram of distribution of posterior probabilities for AncSR2 and posterior probabilities of amino acid residues lining the binding pocket.
