# Supplementary material for: Evolution of Minimal Specificity and Promiscuity in Steroid Hormone Receptors
Source: PLoS Genet. 2012 Nov 15;8(11):e1003072. doi: 10.1371/journal.pgen.1003072 (PMC3499368; doi:10.1371/journal.pgen.1003072)
Supplement: Figure S2 — Histogram of distribution of posterior probabilities for AncSR1 and posterior probabilities of amino acid residues lining the binding pocket. (PDF) [file pgen.1003072.s002.pdf]

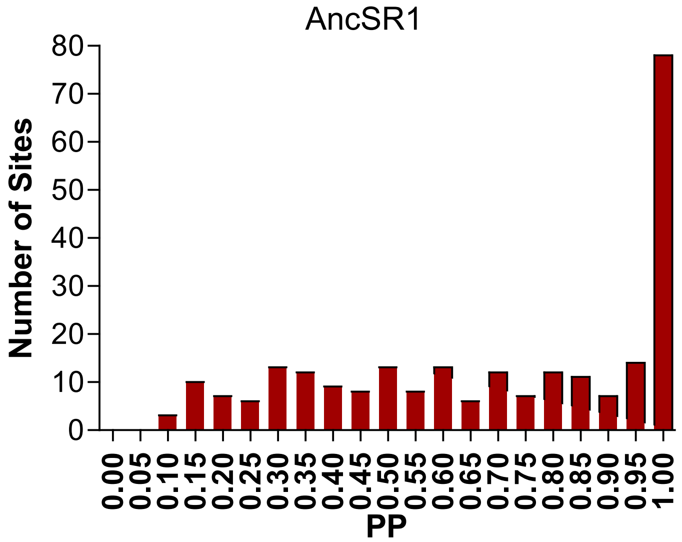

AncSR1 binding pocket

Position	Reconstructed Amino Acid	Probability	Alt. State #1	Probability
41	**E**	1.00		
75	**L**	1.00		
37	**L**	1.00		
38	**A**	1.00		
71	**W**	1.00		
34	**L**	0.99	I	0.00
225	**L**	1.00	M	0.00
35	**T**	0.97	S	0.02
31	**M**	0.62	L	0.27
221	**L**	0.84	M	0.10
219	**V**	1.00		
207	**L**	0.97	F	0.03
210	**M**	0.47	I	0.44
206	**H**	1.00	Y	0.00
203	**G**	0.88	A	0.11
110	**M**	0.98	L	0.02
113	**I**	0.99	L	0.00
72	**M**	0.77	L	0.23
117	**I**	0.79	V	0.16
76	**I**	0.50	M	0.46
79	**L**	0.99	M	0.01
94	**F**	1.00		
82	**R**	1.00		
**Mean Posterior Probability**		0.90		

Fig. S2 Histogram of distribution of posterior probabilities for AncSR1 and posterior probabilities of amino acid residues lining the binding pocket. The numbers next to the residues indicate the position of the residue in the binding pocket (Fig. 1C).
