# Supplementary material for: Evolution of Minimal Specificity and Promiscuity in Steroid Hormone Receptors
Source: PLoS Genet. 2012 Nov 15;8(11):e1003072. doi: 10.1371/journal.pgen.1003072 (PMC3499368; doi:10.1371/journal.pgen.1003072)
Supplement: Figure S3 — Representative dose activation curves of AncSR1 in response to cholesterol and a library of hormones (#0-23). (PDF) [file pgen.1003072.s003.pdf]

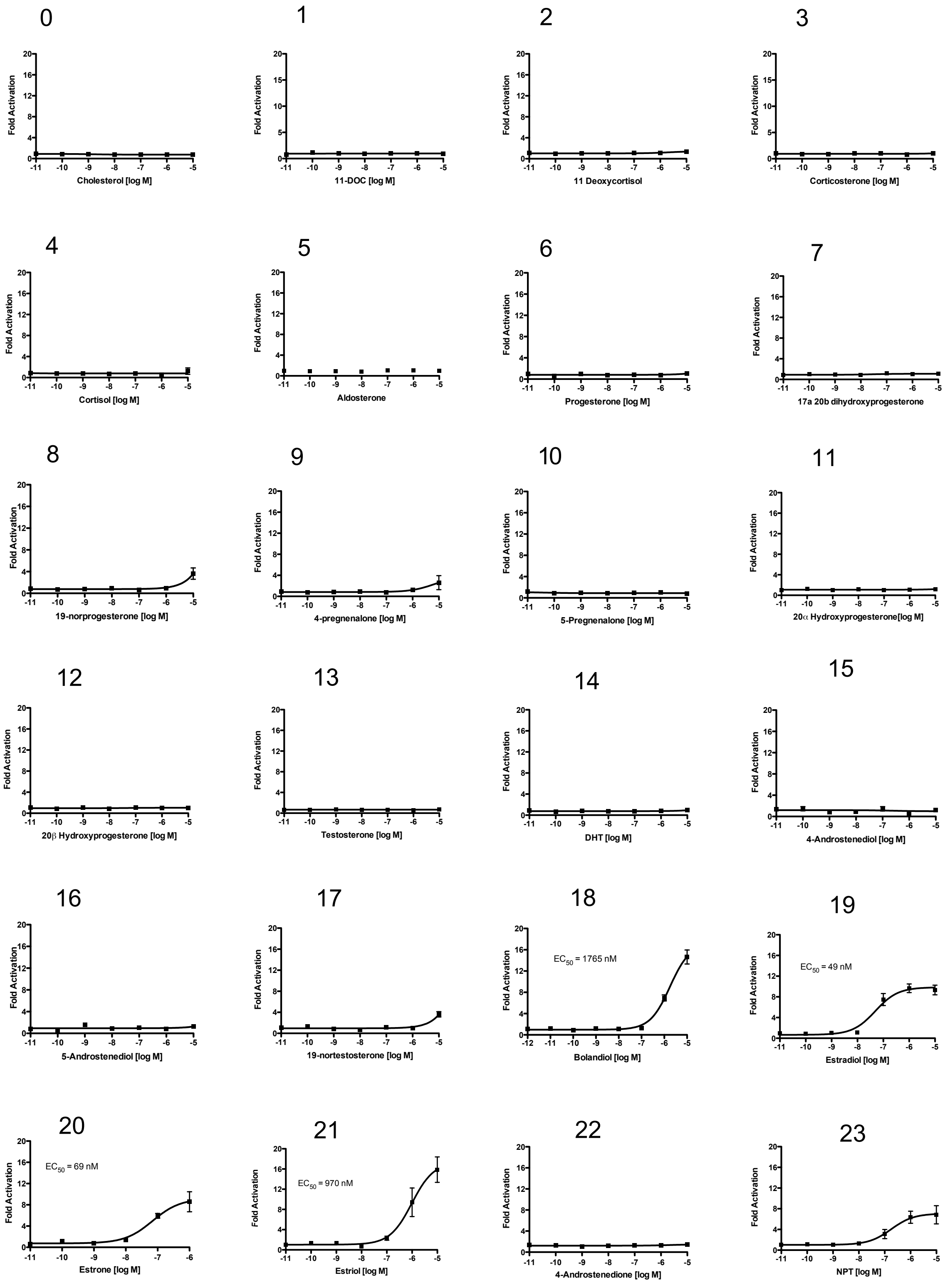

Fig. S3 Representative dose activation curves of AncSR1 in response to cholesterol and a library of hormones (#0-23). Activation is shown as the fold activation of a luciferase construct above vehicle-only (ethanol) treatment. Numbers above graphs correspond to the numbers indicated in Fig. 2A. Pubmed compound identifier numbers are provided in Table S4. Error bars indicate SEM.
