# Supplementary material for: Evolution of Minimal Specificity and Promiscuity in Steroid Hormone Receptors
Source: PLoS Genet. 2012 Nov 15;8(11):e1003072. doi: 10.1371/journal.pgen.1003072 (PMC3499368; doi:10.1371/journal.pgen.1003072)
Supplement: Figure S5 — The specificity of AncSR1 is robust to uncertainty in the reconstruction. (PDF) [file pgen.1003072.s005.pdf]

A

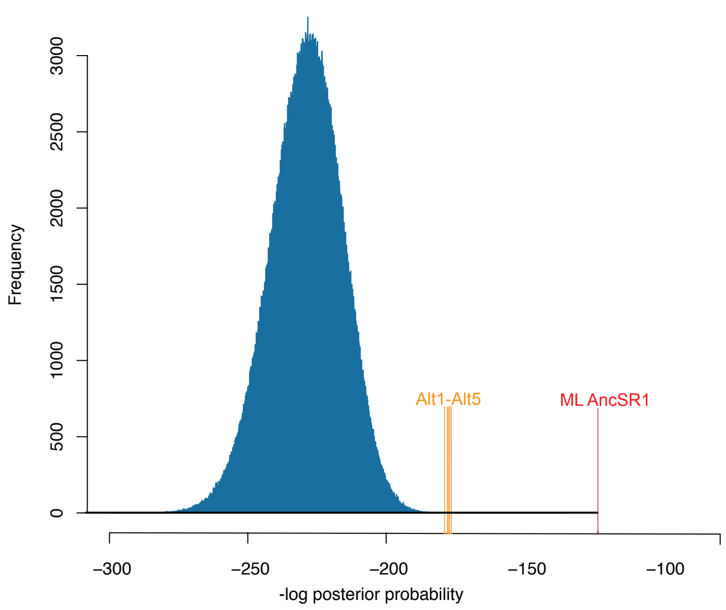

	Ln posterior probability of sequence (LnPP)	LnPP - LnPP of ML reconstruction	Mean PP over sites	# Sites different from ML AncSR1 total sites
**ML AncSR1**	**-122.894**	**-**	**0.69**	**0/249**
AncSR1_Alt1	-176.493	53.599	0.64	57/249
AncSR1_Alt2	-177.222	54.328	0.65	55/249
AncSR1_Alt3	-177.436	54.542	0.64	57/249
AncSR1_Alt4	-177.693	54.799	0.65	55/249
AncSR1_Alt5	-178.833	55.939	0.65	59/249

Position in pocket (Fig. 1C)	Reconstructed Amino Acid	Probability	Alt. State #1	Probability	Alt 1	Alt 2	Alt3	Alt 4	Alt 5
41	**E**	1.00			.	.	.	.	.
75	**L**	1.00			.	.	.	.	.
37	**L**	1.00			.	.	.	.	.
38	**A**	1.00			.	.	.	.	.
71	**W**	1.00			.	.	.	.	.
34	**L**	0.99	I	0.00	.	.	.	.	.
225	**L**	1.00	M	0.00	.	.	.	.	.
35	**T**	0.97	S	0.02	.	.	.	.	.
31	**M**	0.62	L	0.27	.	.	.	.	.
221	**L**	0.84	M	0.10	.	.	.	.	.
219	**V**	1.00			.	.	.	.	.
207	**L**	0.97	F	0.03	.	.	.	.	.
210	**M**	0.47	I	0.44	**I**	.	**I**	.	**I**
206	**H**	1.00	Y	0.00	.	.	.	.	.
203	**G**	0.88	A	0.11	.	.	.	.	.
110	**M**	0.98	L	0.02	.	.	.	.	.
113	**I**	0.99	L	0.00	.	.	.	.	.
72	**M**	0.77	L	0.23	.	.	**L**	.	.
117	**I**	0.79	V	0.16	.	.	.	**V**	**V**
76	**I**	0.50	M	0.46	**M**	.	**M**	.	**M**
79	**L**	0.99	M	0.01	.	.	.	.	.
94	**F**	1.00			.	.	.	.	.
82	**R**	1.00			.	.	.	.	.

B

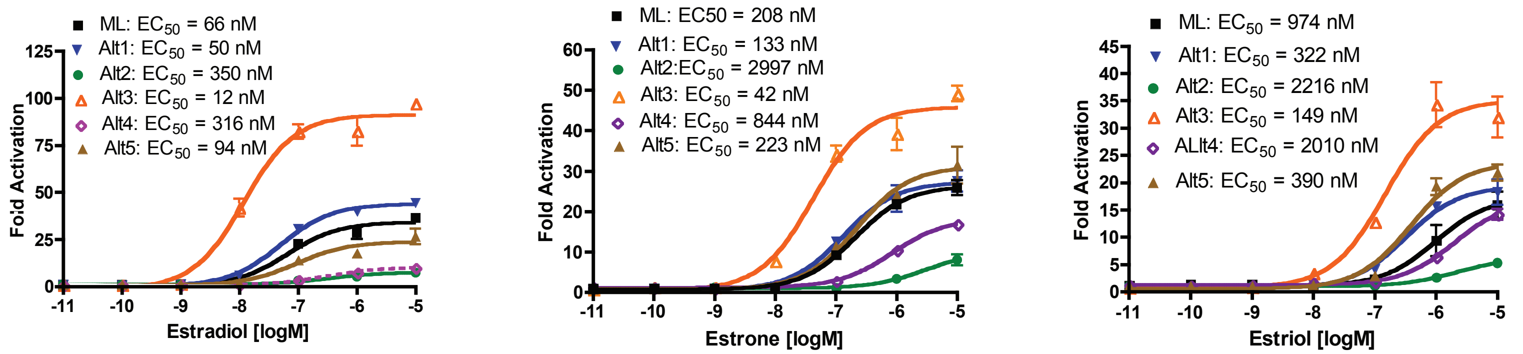

C

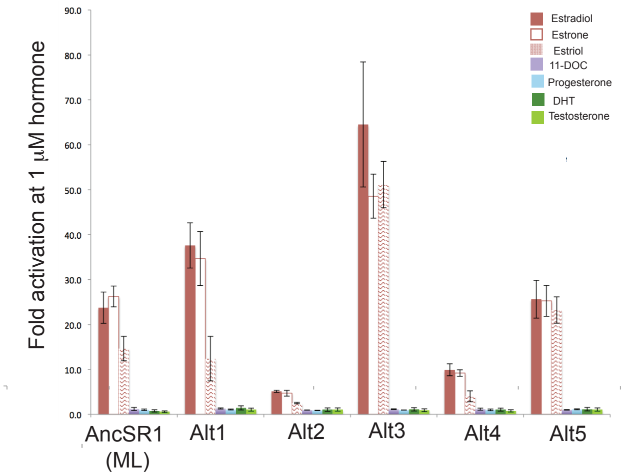

Figure S5.The ligand specificity of AncSR1 is robust to uncertainty in the reconstruction. A) Distribution of posterior probabilities of 1 million protein sequences assembled by by sampling amino acid states at each site from the posterior probability distribution for AncSR1 LBD. The ML reconstruction (AncSR1-ML) and the five alternative sequences with the highest posterior probability (Alt1-Alt5) in the 1 million samples are indicated on the graph. The top table shows the posterior probability (PP) of each sequence in natural logarithmic units, the reduction in posterior probability compared to AncSR1-ML, the PP averaged over sites, and the number of sites with amino acids different from ML-AncSR1. The table below shows the binding pocket residues and their states in the five alternate ancestors. A dot indicates the same state as AncSR1. B) AncSR1-ML and all five alternate ancestors are activated in a dose-responsive manner by estrogens, but C) none are activated by 11-deoxycorticosterone (11-DOC), progesterone, dihydrotestosterone (DHT), or testosterone. The fold activation at 10^{-6} M is shown. The column height and error bars indicate the mean and SEM of three independent experiments, respectively.
