# Supplementary material for: Evolution of Minimal Specificity and Promiscuity in Steroid Hormone Receptors
Source: PLoS Genet. 2012 Nov 15;8(11):e1003072. doi: 10.1371/journal.pgen.1003072 (PMC3499368; doi:10.1371/journal.pgen.1003072)
Supplement: Figure S6 — The specificity of AncSR2 is robust to uncertainty in the reconstruction. (PDF) [file pgen.1003072.s006.pdf]

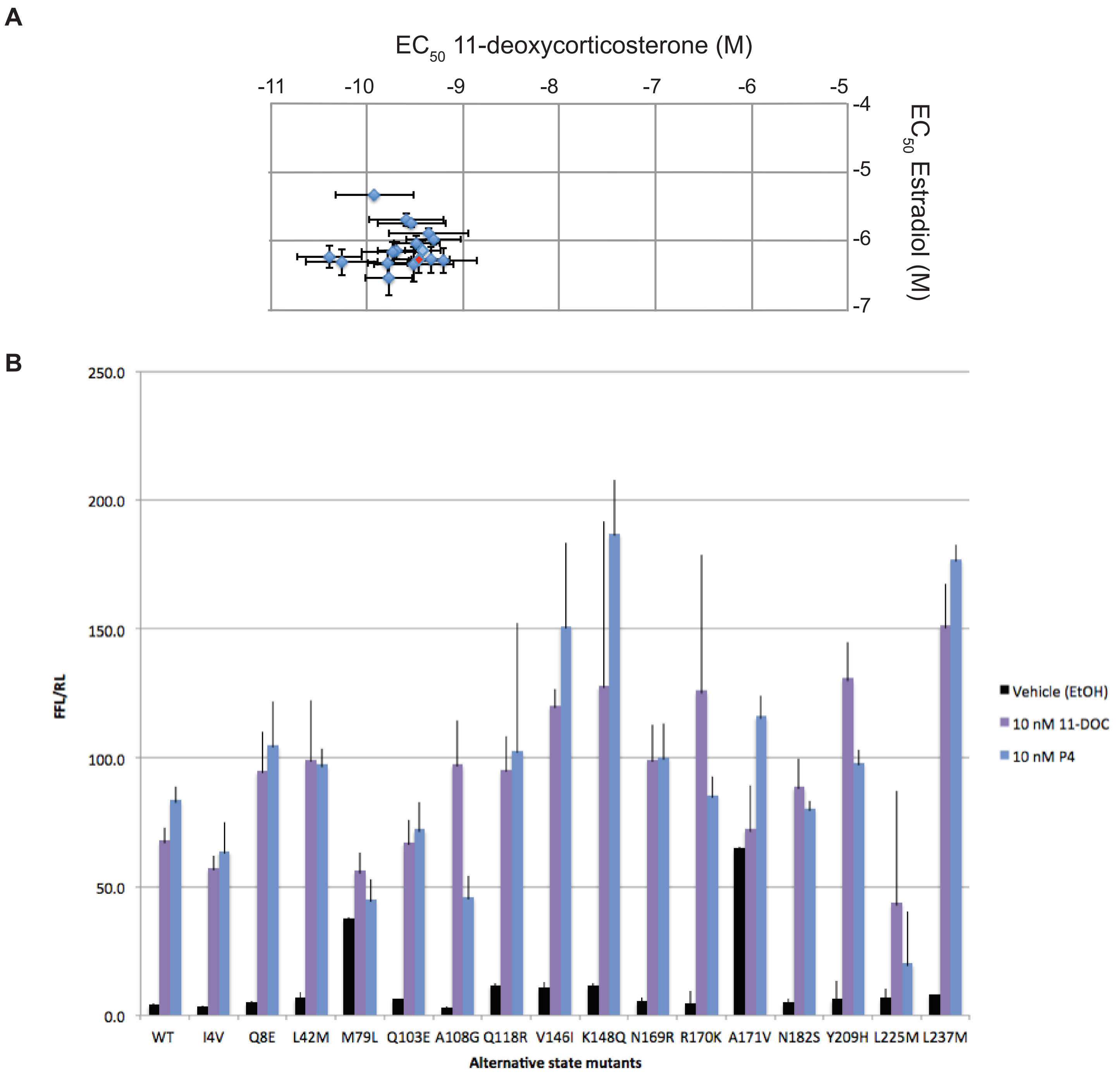

Fig. S6 **A**. Sensitivity of AncSR2 alternative state mutants to 11-deoxycorticosterone (11-DOC) and estradiol. The ML Met79 AncSR2 ancestor is indicated in red. **B**. Fold activation of the AncSR2 alternative state mutants by 1% ethanol (EtOH; vehicle control), 10 nM 11-DOC, and 10 nM progesterone (P4). The M79L and A171V AncSR2 mutants are constitutively active.
