# Supplementary material for: Evolution of Minimal Specificity and Promiscuity in Steroid Hormone Receptors
Source: PLoS Genet. 2012 Nov 15;8(11):e1003072. doi: 10.1371/journal.pgen.1003072 (PMC3499368; doi:10.1371/journal.pgen.1003072)
Supplement: Figure S7 — Sensitivities of extant human receptors to an estrogen, androgen, progestagen, and corticosteroid. (PDF) [file pgen.1003072.s007.pdf]

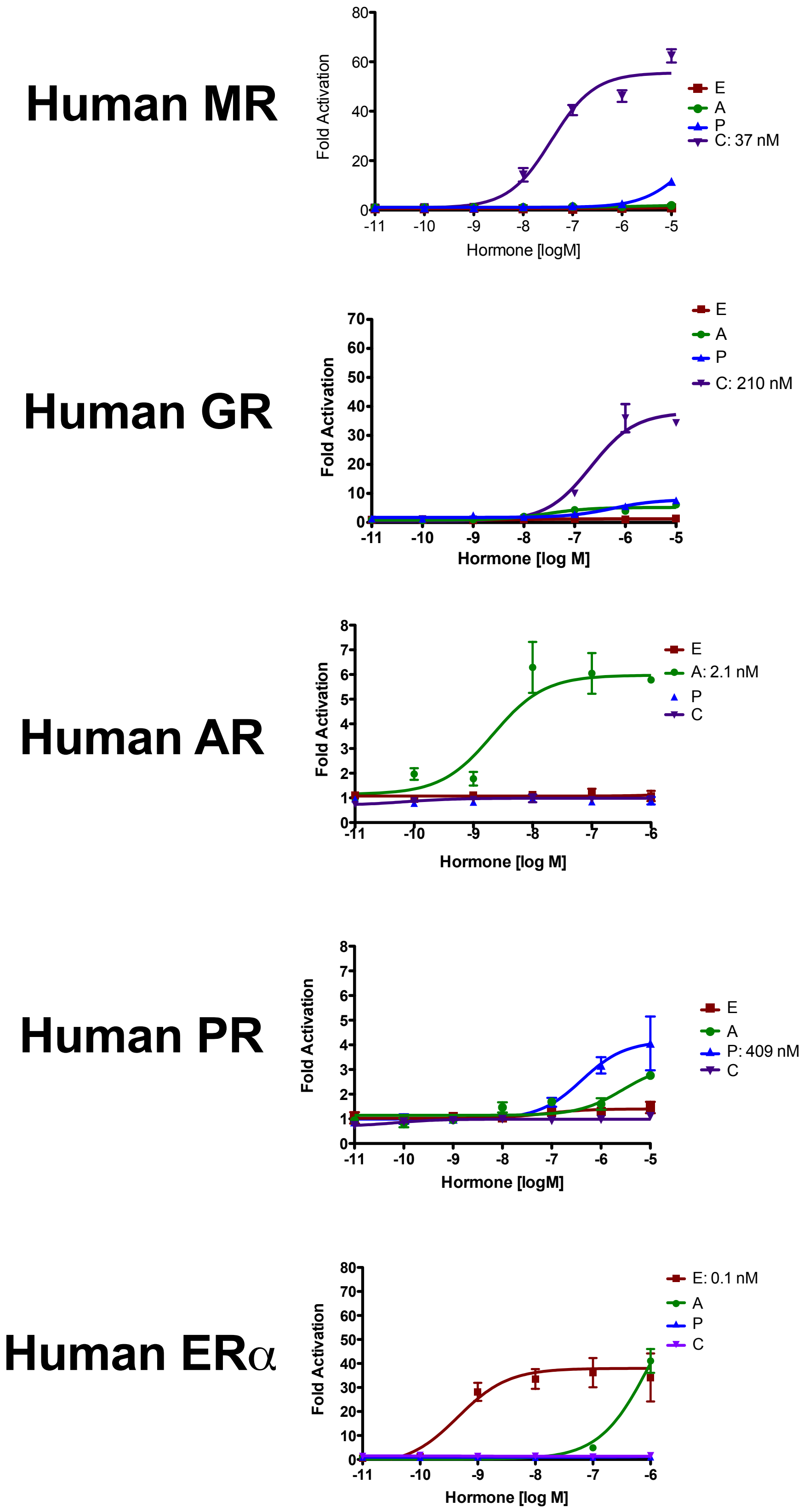

Fig. S7. Sensitivity of the ligand binding domains of the mineralocorticoid receptor (MR), glucocorticoid receptor (GR), androgen receptor (AR), progesterone receptor (PR), and human estrogen receptor alpha (hERα) to a representative estrogen (**E**, estradiol), androgen (**A**, dihydrotestosterone), progestagen (**P**, progesterone), and corticsteroid (**C**, cortisol). Only EC50 values < 1000 nM are indicated.
