# Supplementary material for: Evolution of Minimal Specificity and Promiscuity in Steroid Hormone Receptors
Source: PLoS Genet. 2012 Nov 15;8(11):e1003072. doi: 10.1371/journal.pgen.1003072 (PMC3499368; doi:10.1371/journal.pgen.1003072)
Supplement: Figure S8 — Activation of the estrogen receptor ligand binding domains of two annelids and human ERα. (PDF) [file pgen.1003072.s008.pdf]

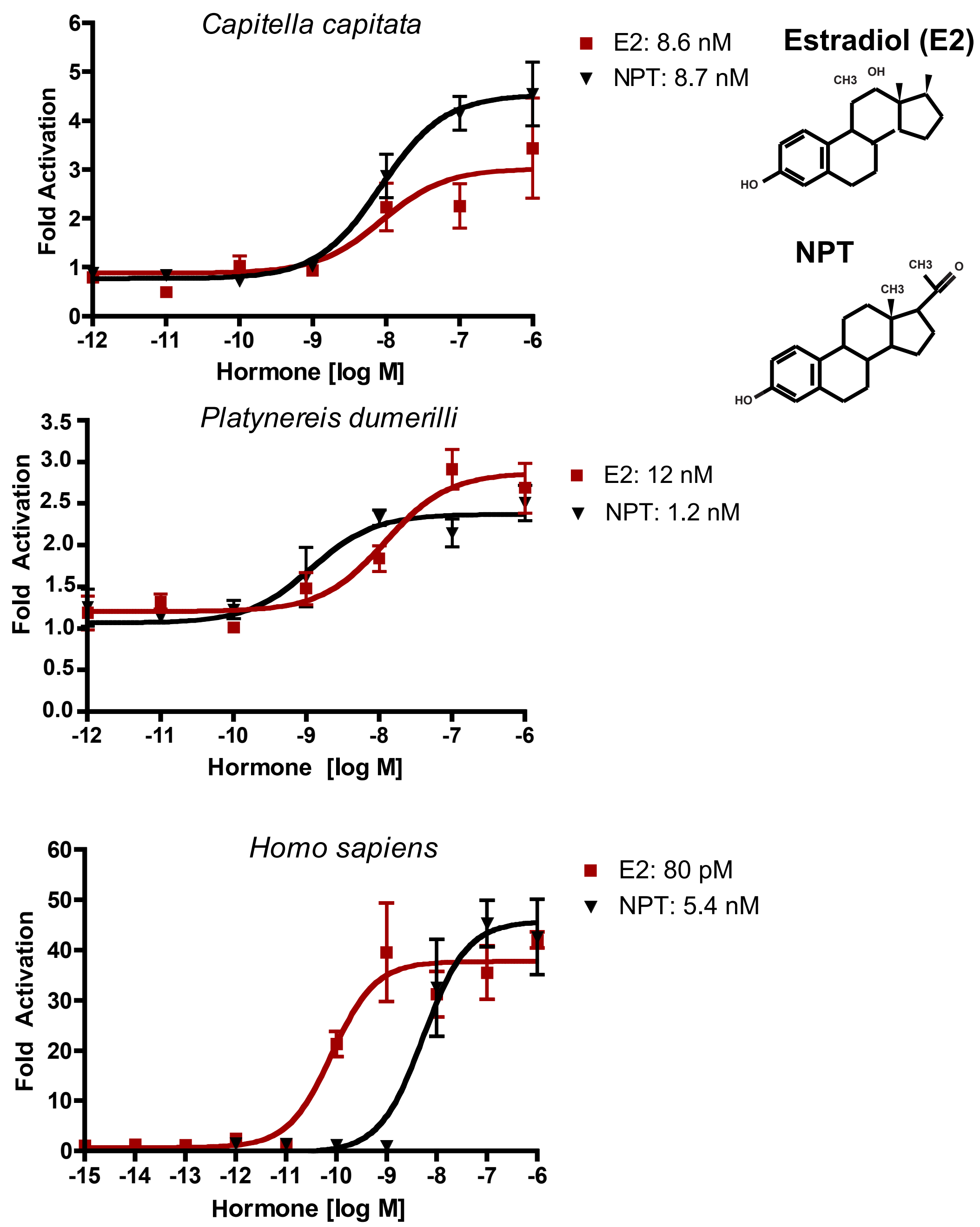

Fig. S8 Activation of the estrogen receptor ligand binding domains of two annelids (*Capitella capitata*, a polychaete worm & *Platynereis dumerilii*, a marine worm) and human ERα by estradiol and the synthetic steroid NPT, which has an aromatized A-ring & progesterone-like D-ring. Values adjacent to the hormone legends (red squares and black triangles) indicate the EC_{50} value for that hormone.
