# Supplementary material for: Evolution of Minimal Specificity and Promiscuity in Steroid Hormone Receptors
Source: PLoS Genet. 2012 Nov 15;8(11):e1003072. doi: 10.1371/journal.pgen.1003072 (PMC3499368; doi:10.1371/journal.pgen.1003072)
Supplement: Figure S9 — AncSR2 is not activated by the nonsteroidal ER agonists diethylstilbestrol and genistein and is not inhibited by ICI182870 and 4-hydroxytamoxifen. (PDF) [file pgen.1003072.s009.pdf]

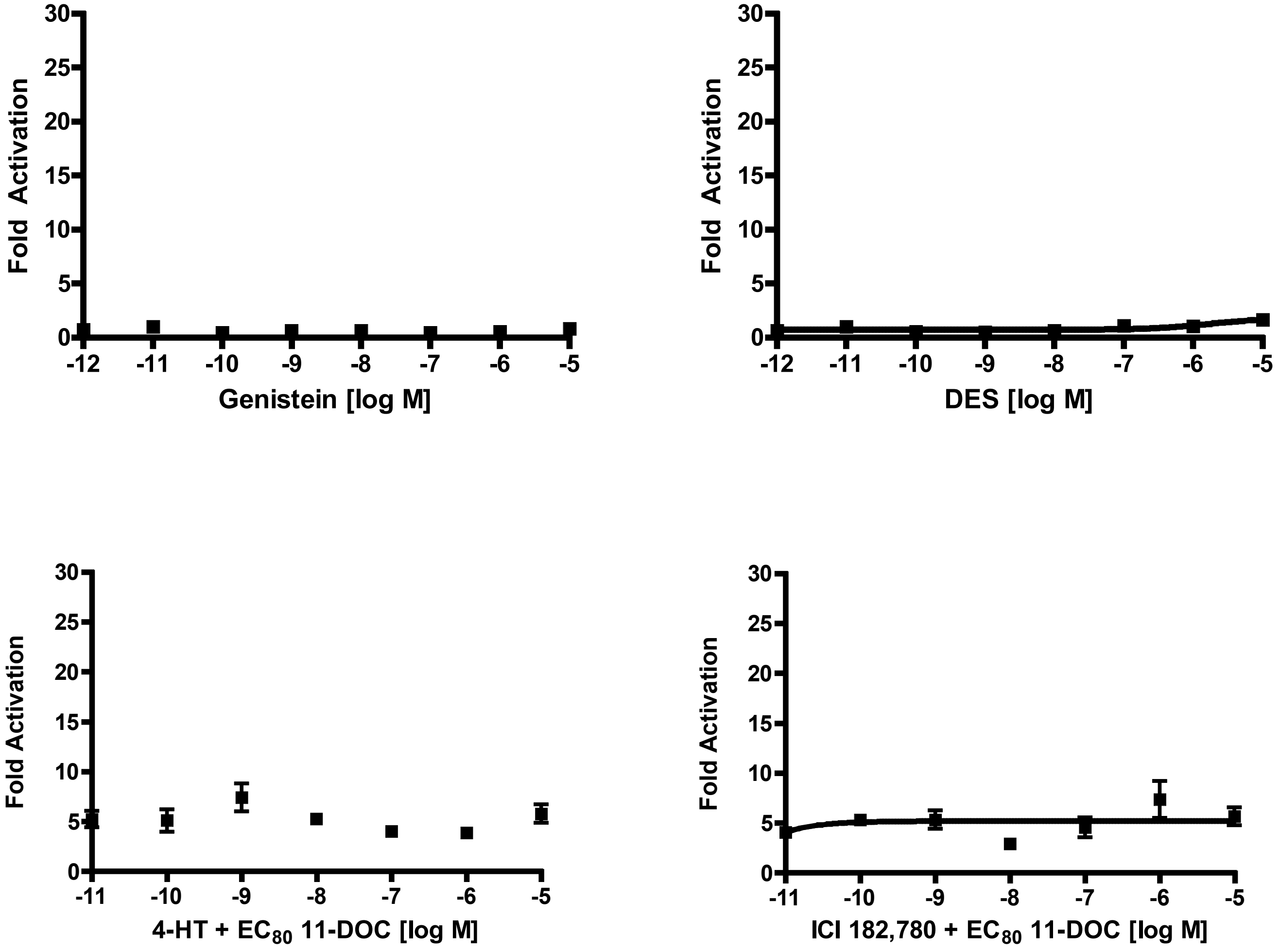

Fig. S9 AncSR2 is not activated by the nonsteroidal ER agonists diethylstilbestrol (DES) and genistein and is not inhibited by 4-hydroxytamoxifen (4-HT) or ICI1 82,780 in the presence of EC_{80} 11-deoxycorticosterone (1.7 nM).
