# Supplementary material for: Evolution of Minimal Specificity and Promiscuity in Steroid Hormone Receptors
Source: PLoS Genet. 2012 Nov 15;8(11):e1003072. doi: 10.1371/journal.pgen.1003072 (PMC3499368; doi:10.1371/journal.pgen.1003072)
Supplement: Figure S10 — Unreduced ML steroid receptor phylogeny based on alignment of 184 steroid receptors and related sequences used to reconstruct AncSR2. (PDF) [file pgen.1003072.s010.pdf]

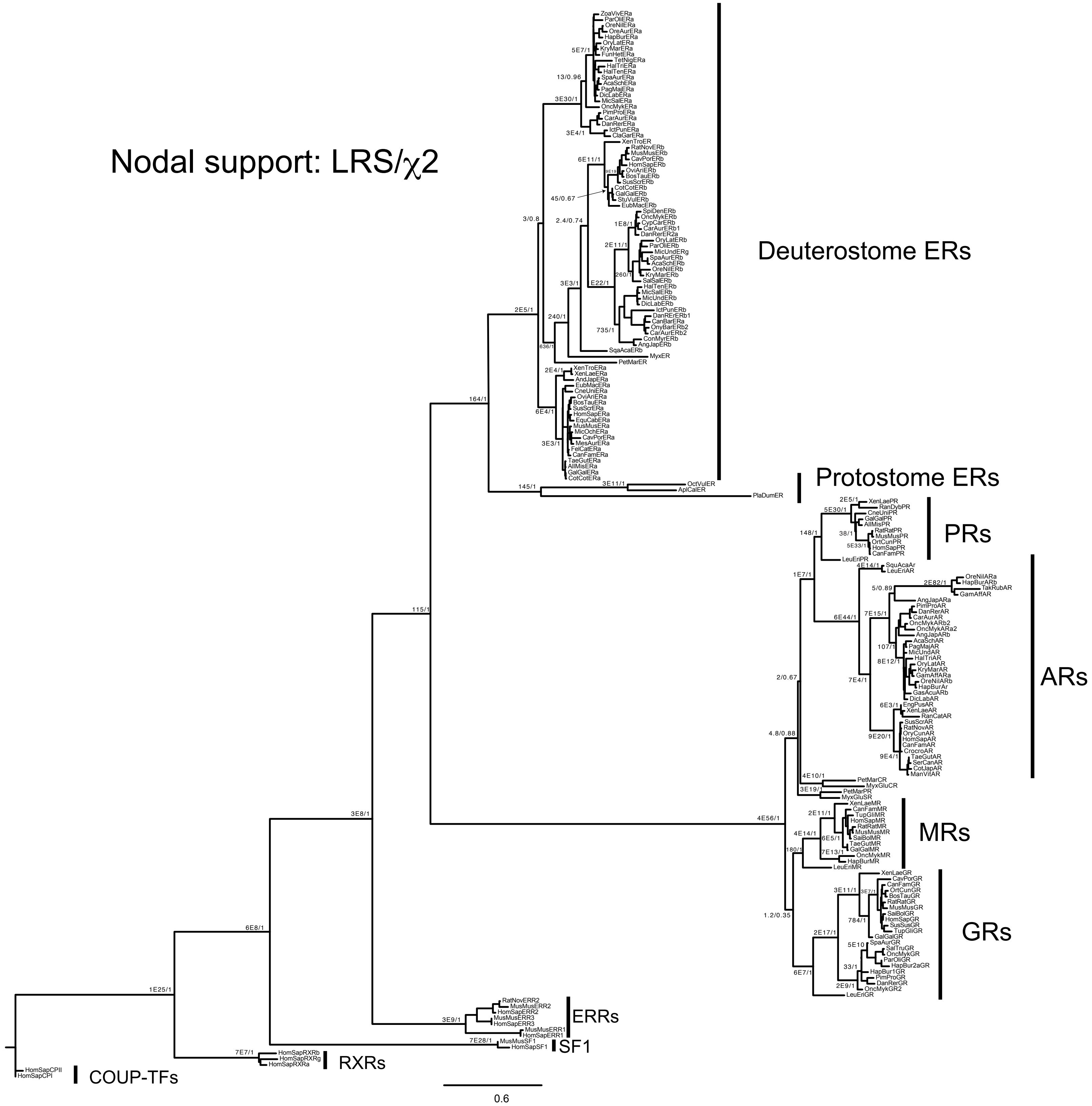

Fig. S10 Unreduced ML steroid receptor phylogeny based on alignment of 184 steroid receptors and related sequences used to reconstruct AncSR2. Nodal support is indicated by likelihood ratio statistics and chi-squared values. ERs, estrogen receptors; PRs, progesterone receptors; ARs, androgen receptors; MRs, mineralocorticoid receptors; GRs, glucocorticoid receptors; ERRs, estrogen-related receptors; SF1, steroidogenic factor 1 receptor; RXR, retinoid X receptors; COUP-TFs, chicken ovalbumin upstream promoter transcription factors.
