# Supplementary figures and images for: Evolution of Minimal Specificity and Promiscuity in Steroid Hormone Receptors
Source: PLoS Genet. 2012 Nov 15;8(11):e1003072. doi: 10.1371/journal.pgen.1003072 (PMC3499368; doi:10.1371/journal.pgen.1003072)

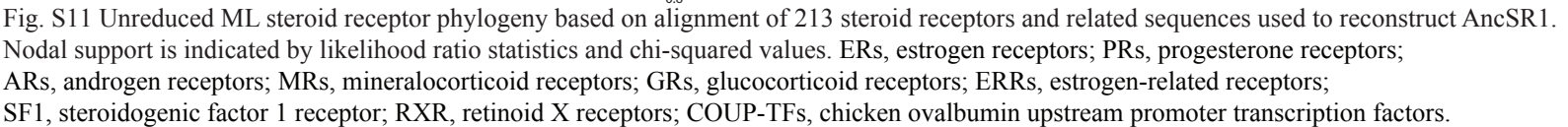

Supplement: Figure S11 — Unreduced ML steroid receptor phylogeny based on alignment of 213 steroid receptors and related sequences used to reconstruct AncSR1. (PDF) [file pgen.1003072.s011.pdf]
