# Supplementary material for: Evolution of Minimal Specificity and Promiscuity in Steroid Hormone Receptors
Source: PLoS Genet. 2012 Nov 15;8(11):e1003072. doi: 10.1371/journal.pgen.1003072 (PMC3499368; doi:10.1371/journal.pgen.1003072)
Supplement: Figure S12 — Unreduced 184-taxon steroid receptor gene duplication phylogeny. (PDF) [file pgen.1003072.s012.pdf]

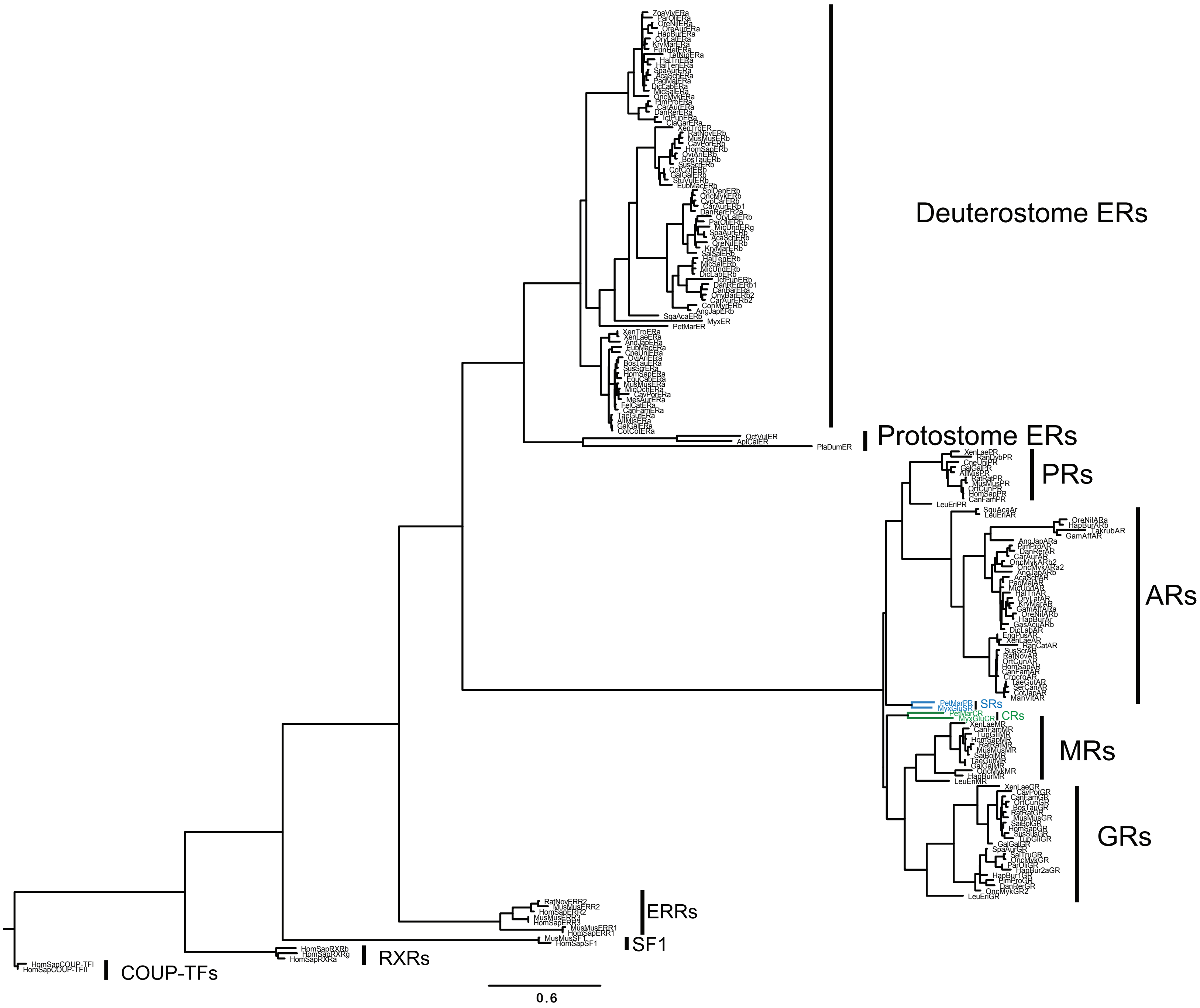

Fig. S12 Unreduced 184-taxon steroid receptor phylogeny with the gnathostome steroid receptor sequences rearranged relative to the ML tree (Fig. S10) to yield a tree that minimizes the number of gene duplication events (gene duplication tree). The gnathostome steroid recpetors (SRs) are indicated in blue and the gnathostome corticoid receptors (CRs) are indicated in green. ERs, estrogen receptors; PRs, progesterone receptors; ARs, androgen receptors; MRs, mineralocorticoid receptors; GRs, glucocorticoid receptors; ERRs, estrogen-related receptors; SF1, steroidogenic factor 1 receptor; RXR, retinoid X receptors; COUP-TFs, chicken ovalbumin upstream promoter transcription factors.
