# Supplementary material for: Evolution of Minimal Specificity and Promiscuity in Steroid Hormone Receptors
Source: PLoS Genet. 2012 Nov 15;8(11):e1003072. doi: 10.1371/journal.pgen.1003072 (PMC3499368; doi:10.1371/journal.pgen.1003072)
Supplement: Figure S13 — Omit maps showing that progesterone and 11-deoxycorticosterone bind directly to AncSR2 to promote receptor activation. (PDF) [file pgen.1003072.s013.pdf]

A

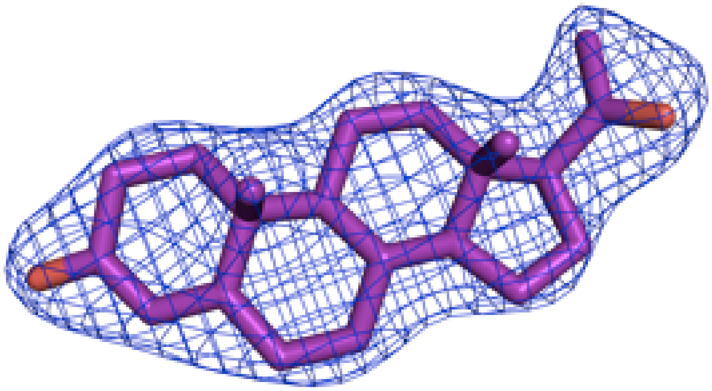

B

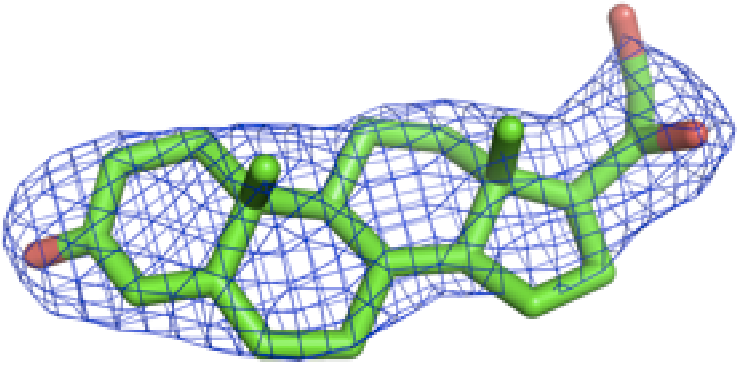

Fig. S13. Omit maps showing that progesterone and 11-deoxycorticosterone bind directly to AncSR2 to promote receptor activation. A. Fo − Fc omit electron Density (blue, contoured at 1 σ) for the bound progesterone (purple, oxygen - red). B. Fo − Fc omit electron density (blue, contoured at 1 σ) for the bound deoxycorticosterone (green, oxygen - red).
