# Supplementary material for: Evolution of Minimal Specificity and Promiscuity in Steroid Hormone Receptors
Source: PLoS Genet. 2012 Nov 15;8(11):e1003072. doi: 10.1371/journal.pgen.1003072 (PMC3499368; doi:10.1371/journal.pgen.1003072)
Supplement: Table S1 — The reconstructed sequence of AncSR2. (PDF) [file pgen.1003072.s014.pdf]

Table S1. The reconstructed sequence of AncSR2. Ambiguously reconstructed sites with a posterior probability (PP) > 0.2 (grey shading) were introduced into the ML ancestor and their responses to 11-deoxycorticosterone and estradiol evaluated using a luciferase reporter assay.

AncSR2 reconstructed on 184-taxa ML tree

Residue No.	Reconstructed Amino Acid	PP	Alt state 1	PP	Alt state 2	PP
1	P	1.00				
2	S	0.81	T	0.19	P	0.01
3	L	0.91	I	0.04	M	0.03
4	I	0.78	V	0.21	M	0.00
5	S	0.94	T	0.05	A	0.01
6	I	0.94	V	0.03	L	0.02
7	L	1.00				
8	Q	0.50	E	0.49	K	0.01
9	A	0.56	V	0.19	S	0.13
10	I	1.00				
11	E	1.00				
12	P	1.00				
13	E	0.99	D	0.01		
14	V	0.96	I	0.04	M	0.00
15	V	0.98	I	0.02	L	0.00
16	Y	1.00	F	0.00		
17	A	1.00				
18	G	1.00				
19	Y	1.00	F	0.00		
20	D	1.00				
21	N	0.81	S	0.19	T	0.00
22	T	0.61	S	0.38	A	0.01
23	Q	0.97	R	0.02	H	0.00
24	P	1.00				
25	D	0.95	E	0.04	M	0.00
26	T	1.00				
27	T	0.99	S	0.01	A	0.00
28	N	0.97	S	0.02	D	0.01
29	Y	0.80	H	0.19	R	0.01
30	L	1.00	M	0.00		
31	L	1.00				
32	S	0.90	T	0.10	A	0.00
33	S	1.00	T	0.00		
34	L	1.00				
35	N	1.00				
36	R	0.99	K	0.01	Q	0.00
37	L	1.00				
38	A	0.87	C	0.06	G	0.05
39	E	0.85	G	0.13	D	0.02
40	K	0.61	R	0.40		
41	Q	1.00				
42	L	0.77	M	0.23		
43	V	1.00	I	0.00		
44	S	0.92	R	0.05	P	0.02
45	V	1.00	I	0.00		
46	V	1.00				
47	K	1.00	R	0.00		
48	W	1.00				
49	A	1.00				
50	K	1.00				
51	A	0.88	V	0.08	S	0.04
52	L	1.00	M	0.00		
53	P	1.00				
54	G	1.00				
55	F	1.00				
56	R	1.00	K	0.00		
57	N	0.99	S	0.01	D	0.00
58	L	1.00				
59	H	0.98	P	0.01	Q	0.01
60	L	0.79	I	0.21	M	0.00
61	D	0.99	E	0.01	N	0.00
62	D	1.00				
63	Q	1.00				
64	M	1.00				
65	T	1.00				
66	L	1.00				
67	I	0.52	L	0.48	M	0.00
68	Q	1.00				
69	Y	1.00				
70	S	1.00				
71	W	1.00				
72	M	1.00				
73	G	0.75	S	0.14	C	0.10
74	L	1.00				
75	M	1.00				
76	A	0.88	S	0.05	T	0.03
77	F	1.00				
78	A	0.84	S	0.15	G	0.00
79	L	0.59	M	0.41	I	0.00
80	G	0.92	S	0.07	A	0.02
81	W	1.00				
82	R	1.00				
83	S	1.00				

Residue No.	Reconstructed Amino Acid	PP	Alt state 1	PP	Alt state 2	PP
84	Y	1.00				
85	K	0.99	R	0.01	Q	0.00
86	H	1.00				
87	T	0.82	A	0.07	V	0.06
88	N	1.00				
89	G	1.00	S	0.00		
90	Q	0.76	K	0.13	N	0.04
91	M	1.00	L	0.00		
92	L	1.00				
93	Y	1.00	F	0.00		
94	F	1.00				
95	A	1.00				
96	P	1.00				
97	D	1.00				
98	L	1.00				
99	I	0.98	V	0.02		
100	F	1.00				
101	N	1.00				
102	E	1.00				
103	Q	0.63	E	0.36	D	0.01
104	R	1.00				
105	M	1.00				
106	Q	0.99	H	0.00	K	0.00
107	Q	1.00				
108	S	1.00				
109	A	1.00	T	0.00	S	0.00
110	M	1.00				
111	Y	1.00	F	0.00	H	0.00
112	D	0.73	E	0.27	N	0.00
113	L	1.00				
114	C	1.00				
115	Q	0.48	L	0.18	M	0.09
116	G	1.00				
117	M	1.00				
118	Q	0.48	R	0.48	H	0.02
119	Q	0.99	K	0.01	N	0.00
120	I	1.00	V	0.00		
121	S	0.99	A	0.01	T	0.00
122	Q	0.47	L	0.11	K	0.09
123	E	1.00	D	0.00		
124	F	1.00				
125	V	0.87	I	0.07	M	0.03
126	R	0.98	K	0.02	H	0.00
127	L	1.00				
128	Q	1.00				
129	V	0.97	L	0.03	I	0.01
130	T	0.98	S	0.02	A	0.00
131	Q	0.53	H	0.28	Y	0.17
132	E	1.00	D	0.01		
133	E	1.00				
134	F	0.99	Y	0.01		
135	L	1.00				
136	C	1.00				
137	M	1.00				
138	K	1.00				
139	A	0.87	V	0.13		
140	L	0.98	I	0.02	M	0.00
141	L	1.00				
142	L	1.00				
143	L	1.00				
144	S	0.99	N	0.01	G	0.00
145	T	1.00				
146	V	0.67	I	0.33		
147	P	1.00				
148	K	0.61	Q	0.34	R	0.04
149	E	0.54	D	0.46	N	0.00
150	G	1.00				
151	L	1.00				
152	K	1.00	R	0.00		
153	S	1.00	T	0.00	N	0.00
154	Q	1.00	H	0.00		
155	A	0.83	T	0.13	S	0.04
156	S	0.34	Y	0.17	A	0.15
157	F	1.00				
158	D	0.95	E	0.05	N	0.00
159	E	1.00	D	0.00	Q	0.00
160	M	0.99	I	0.01	L	0.00
161	R	1.00				
162	M	1.00	I	0.00	L	0.00
163	N	0.98	S	0.02	D	0.00
164	Y	1.00				
165	I	1.00				
166	K	0.68	R	0.32	Q	0.00

Residue No.	Reconstructed Amino Acid	PP	Alt state 1	PP	Alt state 2	PP
167	E	1.00				
168	L	1.00				
169	N	0.43	R	0.25	G	0.18
170	R	0.66	K	0.34	Q	0.00
171	A	0.68	V	0.31	T	0.01
172	I	0.94	V	0.06		
173	A	0.68	V	0.20	G	0.05
174	K	0.55	R	0.34	Q	0.06
175	K	0.55	R	0.24	Q	0.14
176	E	0.98	D	0.02	Q	0.00
177	N	0.85	K	0.08	G	0.04
178	N	1.00	S	0.00	D	0.00
179	S	0.53	T	0.24	A	0.16
180	A	0.47	G	0.24	S	0.19
181	Q	0.99	E	0.01		
182	N	0.56	S	0.41	G	0.01
183	W	1.00				
184	Q	1.00	H	0.00		
185	R	1.00				
186	F	1.00				
187	Y	1.00				
188	Q	1.00				
189	L	1.00				
190	T	1.00				
191	K	1.00				
192	L	0.99	M	0.01		
193	L	1.00				
194	D	1.00				
195	S	1.00				
196	M	1.00				
197	H	1.00				
198	D	0.97	E	0.03		
199	L	1.00	M	0.00		
200	V	1.00				
201	G	1.00	E	0.00	S	0.00
202	G	0.93	K	0.05	N	0.01
203	L	1.00				
204	L	1.00				
205	Q	0.99	E	0.01		
206	F	1.00				
207	C	1.00				
208	F	1.00				
209	Y	0.60	H	0.30	N	0.07
210	T	1.00				
211	F	1.00				
212	V	0.99	I	0.01	M	0.01
213	Q	0.90	E	0.10		
214	S	1.00	N	0.00		
215	Q	0.82	K	0.17	R	0.01
216	A	0.95	T	0.03	S	0.01
217	L	1.00	M	0.00		
218	S	0.96	K	0.01	N	0.01
219	V	1.00				
220	E	1.00				
221	F	1.00				
222	P	1.00				
223	E	1.00	D	0.00		
224	M	1.00				
225	L	0.77	M	0.23	I	0.00
226	V	0.63	S	0.18	A	0.14
227	E	1.00				
228	I	1.00	M	0.00		
229	I	1.00				
230	S	1.00	N	0.00	T	0.00
231	A	0.90	D	0.07	N	0.03
232	Q	1.00				
233	L	1.00	M	0.00	F	0.00
234	P	1.00				
235	K	0.95	R	0.05	Q	0.00
236	V	0.86	I	0.14	M	0.00
237	L	0.49	M	0.22	T	0.14
238	A	0.92	S	0.05	T	0.03
239	G	1.00				
240	M	0.97	L	0.01	I	0.01
241	A	0.96	T	0.03	V	0.00
242	K	1.00	R	0.00		
243	P	1.00	S	0.00		
244	L	1.00				
245	L	0.99	F	0.00	M	0.00
246	F	1.00				
247	H	1.00				
248	K	0.91	R	0.07	Q	0.02
249	K	1.00	R	0.00		
	Mean Posterior Probability	0.93				
