# Supplementary material for: Evolution of Minimal Specificity and Promiscuity in Steroid Hormone Receptors
Source: PLoS Genet. 2012 Nov 15;8(11):e1003072. doi: 10.1371/journal.pgen.1003072 (PMC3499368; doi:10.1371/journal.pgen.1003072)
Supplement: Table S2 — The reconstructed sequence of AncSR1. (PDF) [file pgen.1003072.s015.pdf]

Table S2. The reconstructed sequence of AncSR1 and associated posterior probability (PP) values.

AncSR1 reconstructed on 213-taxon gene duplication tree

Residue No.	Reconstructed Amino Acid	PP	Alt state 1	PP	Alt state 2	PP
1	E	0.14	K	0.13	R	0.09
2	K	0.17	R	0.14	S	0.10
3	P	0.21	S	0.12	A	0.10
4	L	0.12	P	0.11	A	0.10
5	S	0.12	K	0.09	A	0.09
6	S	0.13	A	0.09	K	0.08
7	L	0.16	S	0.09	A	0.08
8	P	0.31	S	0.26	T	0.15
9	A	0.38	S	0.15	T	0.13
10	N	0.22	E	0.16	K	0.12
11	Q	0.28	S	0.15	P	0.11
12	L	0.45	I	0.30	V	0.16
13	I	0.62	V	0.32	L	0.03
14	S	0.40	N	0.14	A	0.13
15	A	0.57	S	0.09	T	0.07
16	L	1.00				
17	L	0.67	M	0.13	Q	0.05
18	E	0.47	K	0.16	Q	0.07
19	A	0.99	V	0.01	T	0.00
20	E	1.00				
21	P	1.00				
22	P	0.94	S	0.02	Q	0.01
23	V	0.29	I	0.28	T	0.10
24	L	0.51	V	0.21	I	0.14
25	Y	0.79	H	0.15	F	0.01
26	S	0.49	A	0.44	T	0.06
27	R	0.16	Q	0.14	H	0.11
28	H	0.97	Y	0.03	Q	0.00
29	D	0.99	N	0.01	E	0.00
30	P	0.99	S	0.00	H	0.00
31	S	0.13	A	0.09	D	0.09
32	K	0.12	L	0.12	R	0.11
33	P	1.00				
34	D	0.16	P	0.16	S	0.15
35	T	0.98	S	0.02	A	0.00
36	E	0.75	D	0.17	K	0.03
37	A	0.48	V	0.14	S	0.10
38	H	0.36	N	0.22	S	0.13
39	L	0.50	M	0.37	I	0.07
40	M	0.62	L	0.27	I	0.10
41	T	0.88	A	0.06	S	0.03
42	S	0.81	T	0.18	A	0.01
43	L	0.99	I	0.00	V	0.00
44	T	0.97	S	0.02	I	0.00
45	N	0.37	D	0.29	E	0.14
46	L	1.00				
47	A	1.00				
48	D	1.00	E	0.00	N	0.00
49	R	0.98	K	0.02		
50	E	1.00				
51	L	1.00				
52	V	0.99	I	0.01		
53	H	0.33	G	0.12	D	0.12
54	I	0.52	V	0.36	M	0.09
55	I	0.99	V	0.01		
56	N	0.68	D	0.20	S	0.05
57	W	1.00				
58	A	1.00				
59	K	1.00				
60	K	0.39	R	0.36	H	0.15
61	I	0.96	V	0.03	L	0.01
62	P	1.00				
63	G	1.00				
64	Y	0.64	F	0.36		
65	S	0.82	T	0.12	A	0.04
66	D	0.48	E	0.28	N	0.12
67	L	1.00				
68	S	0.86	P	0.04	A	0.03
69	L	0.99	M	0.01	I	0.00
70	N	0.64	H	0.09	D	0.08
71	D	1.00				
72	Q	1.00				
73	V	1.00	M	0.00		
74	H	0.63	N	0.14	S	0.08
75	L	1.00				
76	L	0.95	I	0.05	M	0.00
77	Q	0.87	E	0.13	R	0.00
78	S	0.61	C	0.35	A	0.02
79	C	0.60	S	0.31	A	0.06
80	W	1.00				
81	M	0.77	L	0.23	I	0.00
82	E	0.98	D	0.02		
83	L	0.69	I	0.16	V	0.14

Residue No.	Reconstructed Amino Acid	PP	Alt state 1	PP	Alt state 2	PP
84	L	1.00				
85	I	0.50	M	0.46	V	0.04
86	L	0.70	I	0.22	V	0.05
87	G	1.00	D	0.00		
88	L	0.99	M	0.01	V	0.00
89	A	0.68	V	0.29	I	0.02
90	W	1.00				
91	R	1.00				
92	S	1.00				
93	M	0.83	I	0.12	L	0.04
94	D	0.42	E	0.37	Q	0.06
95	H	0.98	Y	0.02		
96	E	0.36	K	0.21	Q	0.14
97	G	0.97	D	0.02	N	0.01
98	K	0.80	E	0.08	R	0.07
99	L	1.00	M	0.00		
100	V	0.50	I	0.39	L	0.05
101	F	1.00				
102	A	1.00				
103	P	1.00	S	0.00	A	0.00
104	D	1.00	N	0.00		
105	L	0.99	M	0.01	F	0.00
106	I	0.61	V	0.25	L	0.08
107	L	0.73	M	0.20	F	0.04
108	D	1.00	N	0.00	E	0.00
109	R	0.91	K	0.06	Q	0.02
110	E	0.29	D	0.21	N	0.16
111	Q	0.69	R	0.23	H	0.04
112	S	0.82	G	0.07	A	0.05
113	K	0.59	R	0.37	Q	0.02
114	C	0.68	S	0.09	A	0.05
115	V	0.59	I	0.17	A	0.12
116	A	0.91	S	0.07	T	0.02
117	G	1.00				
118	M	0.98	L	0.02	I	0.00
119	E	0.22	D	0.15	A	0.13
120	E	0.82	D	0.17	Q	0.01
121	I	0.99	L	0.00	V	0.00
122	C	0.74	S	0.17	F	0.06
123	D	0.42	E	0.30	N	0.06
124	Q	0.79	H	0.13	P	0.03
125	I	0.79	V	0.16	M	0.04
126	L	0.91	M	0.03	F	0.02
127	E	0.35	A	0.30	Q	0.14
128	I	0.46	L	0.24	V	0.19
129	A	0.87	S	0.09	T	0.02
130	S	0.17	Q	0.17	R	0.15
131	Q	0.56	R	0.22	K	0.22
132	F	1.00	Y	0.00		
133	R	0.61	K	0.19	Q	0.04
134	E	0.59	Q	0.15	D	0.12
135	L	0.98	F	0.02	I	0.00
136	K	0.37	Q	0.20	R	0.16
137	V	0.43	L	0.25	I	0.24
138	Q	0.37	N	0.15	E	0.12
139	K	0.53	R	0.44	Q	0.03
140	E	0.96	D	0.03	Q	0.01
141	E	1.00				
142	F	0.93	Y	0.07		
143	V	0.95	I	0.02	L	0.02
144	C	1.00				
145	L	1.00				
146	K	1.00	R	0.00		
147	A	1.00				
148	I	0.94	M	0.03	V	0.02
149	T	0.61	A	0.18	V	0.11
150	L	1.00				
151	L	0.45	V	0.43	I	0.10
152	N	1.00	S	0.00		
153	S	0.55	A	0.38	G	0.04
154	G	0.57	S	0.08	A	0.06
155	V	0.33	I	0.15	L	0.15
156	F	0.34	Y	0.20	C	0.12
157	T	0.31	S	0.14	A	0.11
158	F	0.27	S	0.18	L	0.12
159	L	0.30	S	0.13	M	0.06
160	S	0.42	N	0.11	A	0.09
161	S	0.43	A	0.24	T	0.15
162	D	0.25	A	0.25	E	0.19
163	A	0.23	V	0.22	S	0.16
164	K	0.32	E	0.32	R	0.16
165	R	0.32	G	0.16	K	0.14
166	L	1.00	M	0.00	I	0.00

Residue No.	Reconstructed Amino Acid	PP	Alt state 1	PP	Alt state 2	PP
167	E	0.94	D	0.04	Q	0.01
168	D	0.69	N	0.17	E	0.07
169	H	0.16	Y	0.12	F	0.10
170	E	0.21	D	0.19	G	0.08
171	Q	0.38	K	0.34	R	0.19
172	V	0.63	I	0.36	M	0.00
173	Q	0.70	E	0.15	H	0.09
174	K	0.31	Q	0.23	E	0.16
175	L	0.54	I	0.27	M	0.17
176	Q	0.99	R	0.01	H	0.00
177	D	0.61	E	0.26	Q	0.09
178	K	0.19	N	0.18	T	0.15
179	I	0.83	V	0.11	L	0.06
180	T	0.42	H	0.33	N	0.07
181	D	0.78	E	0.22	N	0.00
182	A	1.00				
183	L	1.00				
184	V	0.30	I	0.12	Q	0.10
185	D	0.68	E	0.16	H	0.08
186	T	0.43	A	0.18	S	0.15
187	V	0.30	C	0.25	T	0.16
188	A	0.54	S	0.18	T	0.08
189	K	0.62	R	0.25	Q	0.04
190	S	0.29	R	0.11	N	0.10
191	H	0.84	Y	0.06	Q	0.05
192	P	0.26	L	0.13	S	0.09
193	D	0.26	E	0.25	N	0.14
194	S	0.35	N	0.20	P	0.17
195	P	0.98	S	0.01	A	0.00
196	Q	0.51	E	0.16	R	0.09
197	Q	0.50	R	0.08	K	0.07
198	S	0.48	P	0.12	A	0.08
199	R	0.87	K	0.08	Q	0.02
200	R	1.00				
201	L	0.67	F	0.13	I	0.11
202	A	0.98	S	0.01	G	0.00
203	Q	0.76	K	0.20	R	0.03
204	L	0.98	M	0.01	I	0.01
205	L	1.00	M	0.00		
206	M	0.51	L	0.48	I	0.00
207	L	0.86	I	0.07	M	0.05
208	L	1.00	M	0.00		
209	S	0.77	P	0.23	T	0.00
210	H	0.87	Q	0.11	E	0.00
211	I	0.68	V	0.23	L	0.07
212	R	1.00				
213	Q	0.98	H	0.01	E	0.01
214	V	0.59	I	0.27	M	0.07
215	S	0.98	A	0.02	T	0.00
216	S	0.27	N	0.26	T	0.21
217	K	0.57	R	0.42	Q	0.01
218	G	0.88	A	0.11	S	0.01
219	I	0.76	V	0.17	M	0.06
220	E	0.78	D	0.13	Q	0.06
221	H	1.00	Y	0.00		
222	L	0.97	F	0.03	I	0.00
223	Y	0.91	F	0.08	H	0.01
224	S	0.49	N	0.18	K	0.07
225	M	0.47	I	0.44	V	0.05
226	K	0.96	R	0.04	Q	0.00
227	S	0.37	C	0.15	N	0.10
228	E	0.78	A	0.07	Q	0.07
229	G	0.69	N	0.15	S	0.11
230	R	0.20	K	0.18	A	0.13
231	V	1.00				
232	P	1.00	S	0.00		
233	L	0.84	M	0.10	F	0.03
234	Y	0.70	H	0.26	C	0.01
235	D	0.96	N	0.02	E	0.02
236	L	1.00				
237	L	1.00	M	0.00		
238	L	0.84	S	0.04	M	0.04
239	E	1.00				
240	M	1.00	I	0.00		
241	L	1.00	I	0.00	V	0.00
242	D	0.82	E	0.18	N	0.00
243	A	1.00				
244	Q	0.89	H	0.09	K	0.01
245	T	0.35	S	0.11	N	0.10
246	S	0.41	P	0.37	A	0.06
247	Q	0.20	H	0.14	A	0.13
248	S	0.30	A	0.27	T	0.17
249	P	0.13	S	0.10	A	0.09
	**Mean Posterior Probability**	0.69				
