# Supplementary material for: Evolution of Minimal Specificity and Promiscuity in Steroid Hormone Receptors
Source: PLoS Genet. 2012 Nov 15;8(11):e1003072. doi: 10.1371/journal.pgen.1003072 (PMC3499368; doi:10.1371/journal.pgen.1003072)
Supplement: Table S3 — Percent similarity of the ligand-binding domains of AncSR1 and AncSR2 to those of extant steroid receptors in humans. (PDF) [file pgen.1003072.s016.pdf]

Table S3. Percent similarity of the ligand-binding domains of AncSR1 and AncSR2 to those of extant steroid receptors in humans.

	**AncSR1**	**AncSR2**
**AncSR1**	100%	
hERα	61.80%	24.80%
hERβ	56.70%	25.70%
hAR	25.60%	62.60%
hPR	29.80%	66.50%
hGR	29.80%	64.80%
hMR	29%	71.70%
**AncSR2**	30.70%	100%
