# Supplementary material for: Evolution of Minimal Specificity and Promiscuity in Steroid Hormone Receptors
Source: PLoS Genet. 2012 Nov 15;8(11):e1003072. doi: 10.1371/journal.pgen.1003072 (PMC3499368; doi:10.1371/journal.pgen.1003072)
Supplement: Table S4 — Pubmed compound identifier (CID) numbers for cholesterol and the synthetic and natural steroid hormones tested in this study. (PDF) [file pgen.1003072.s017.pdf]

Table S4. Pubmed compound identifier (CID) numbers for cholesterol and the synthetic and natural steroid hormones tested in this study . For the two substances that a CID number was not available, the Steraloids catalogue number and name are provided.

Number in text	Steroid Name in Text	Abbreviation	Pubmed Compound Identifier Number (CID)	Steraloids Catalogue #	Steraloids name
1	11-Deoxycorticosterone	11-DOC	6166		
2	11-Deoxycortisol		440707		
3	Corticosterone		5753		
4	Cortisol		5754		
5	Aldosterone		5839		
6	Progesterone		5994		
7	17-Hydroxyprogesterone		6238		
8	19-Norprogesterone		228864		
9	4-Pregnenolone		-	Q3540-000	4-PREGNEN-3β-OL-20-ONE
10	5-Pregnenolone		8955		
11	20α Hydroxyprogesterone		440204		
12	20β Hydroxyprogesterone		92747		
13	Testosterone		6013		
14	Dihydrotestosterone	DHT	10635		
15	4-Androstenediol		136297		
16	5-Androstenediol		10634		
17	19-Nortestosterone		9904		
18	Bolandiol		16141		
19	Estradiol		5757		
20	Estrone		5870		
21	Estriol		5756		
22	4-Androstenedione		6128		
23	19-nor-1, 3, 5(10)-pregnatriene-3-ol-20-one	NPT	-	N0600-000	19-NOR-1, 3, 5(10)-PREGNATRIEN-3-OL-20-ONE
0	Cholesterol		5997		
	Diethylstilbestrol	DES	448537		
	Genistein		5280961		
	4-Hydroxytamoxifen		449459		
	ICI 182,780	fulvestrant	104741		
