# Supplementary material for: Evolution of Minimal Specificity and Promiscuity in Steroid Hormone Receptors
Source: PLoS Genet. 2012 Nov 15;8(11):e1003072. doi: 10.1371/journal.pgen.1003072 (PMC3499368; doi:10.1371/journal.pgen.1003072)
Supplement: Table S5 — Fold preferences of AncSR1 and AncSR2 for the hormone pairs indicated in Figure 2. (PDF) [file pgen.1003072.s018.pdf]

Table S5. Fold preferences of AncSR1 and AnSR2 for the hormone pairs indicated in Fig. 2.

		Hormone 1	EC_{50} (nM)	Hormone 2	EC_{50} (nM)	Ratio H1/H2	Ratio H2/H1
**b**	preference for aromatized A-ring (H1/H2)	18	1765	19	49	36.02	0.03
		22	>10 000	20	69	144.93	0.01
		8	>10 000	23	179	55.87	0.02
**c**	preference for non-aromatized A-ring (H1/H2)	23	182	8	1.6	113.75	0.01
		19	>10 000	18	20	500.00	0.00
**d**	preference for 17-acetyl vs. 17-hydroxy group (H1/H2)	13	116	6	5.5	21.09	0.05
		17	69	8	1.6	43.13	0.02
		15	262	9	2.2	119.09	0.01
		16	83	10	3.8	21.84	0.05
		19	>10 000	23	182	54.95	0.02
**e**	preference for 3-hydroxy vs. 3-keto group (H2/H1)	18	20	17	69	0.29	3.45
		9	2.2	6	5.5	0.40	2.50
		15	262	13	116	2.26	0.44
**f**	preference for 21-OH group vs. H group (H2/H1)	1	0.42	6	5.5	0.08	13.10
		2	1.5	7	19	0.08	12.67
**g**	preference for 11-OH vs. H group (H1/H2)	1	0.42	3	0.31	1.35	0.74
		2	1.5	4	9.8	0.15	6.53
**h**	preference for 17a-H group vs. -OH group (H2/H1)	3	0.31	4	9.8	0.03	31.61
		6	5.5	7	19	0.29	3.45
**i**	preference for 19-H vs. 19-methyl group (H2/H1)	8	1.6	6	5.5	0.29	3.44
		17	69	13	116	0.59	1.68
		18	20	15	262	0.08	13.10

1	11-deoxycorticosterone
2	11-deoxycortisol
3	corticosterone
4	cortisol
5	aldosterone
6	progesterone
7	17a-hydroxyprogesterone
8	19-norprogesterone
9	4-pregnenolone
10	5-pregnenolone
11	20a hydroxyprogesterone
12	20b hydroxyprogesterone
13	testosterone
14	dihydrotestosterone
15	4-androstenediol
16	5-androstenediol
17	19-nortestosterone
18	bolandiol
19	estradiol
20	estrone
21	estriol
22	4-androstenedione
23	NPT
