# Supplementary material for: Evolution of Minimal Specificity and Promiscuity in Steroid Hormone Receptors
Source: PLoS Genet. 2012 Nov 15;8(11):e1003072. doi: 10.1371/journal.pgen.1003072 (PMC3499368; doi:10.1371/journal.pgen.1003072)
Supplement: Table S6 — Data collection and refinement statistics for the AncSR2 crystal structure in complex with 11-DOC and progesterone. (PDF) [file pgen.1003072.s019.pdf]

Table S6. Data collection and refinement statistics for the AncSR2 LBD crystal structure in complex with 11-deoxycorticosterone (11-DOC) and progesterone.

**Data Collection and Refinement Statistics**		
	AncSR2-Progesterone	AncSR2-11-DOC
Resolution (Å)	2.75(2.85-2.75)	2.82 (2.92-2.82)
Space Group	$P2_12_12_1$	$C222_1$
Unit Cell Dimensions	53.47, 112.11, 132.85	52.80, 111.62, 130.77
a, b, c (Å)	90, 90, 90	90, 90, 90
α, β, γ (°)		
No. of Reflections	20584	9198
R^a_{sym}	8.9% (44.3%)	7.1% (34.5%)
Completeness	99.4% (96.1%)	92.6% (70.0%)
Ave. Redundancy	6.8 (5.2)	3.9 (3.1)
I/σ	25.1 (3.5)	19.4 (3.1)
Monomers per asymmetric unit (AU)	2	1
No. of protein atoms/AU	4228	2069
No. of ligand atoms/AU	2	2
No. of waters/AU	65	31
$R^b_{working}$ (R^c_{free})	23.3 (29.1)	23.1 (30.6)
Ave. B-factors (Å^2)		
Protein	63.06	72.12
Ligand	55.63	70.04
Water	63.21	68.44
r.m.s. deviations		
Bond lengths, Å	0.014	0.009
Bond angles, °	1.792	1.298

[a] $R_{sym} = \Sigma|I - \langle I\rangle| / \Sigma|I|$, where I is the observed intensity and <I> is the average intensity of several symmetry-related observations.

[b] $R_{working} = \Sigma||Fo|-|Fc|| / \Sigma|Fo$, where Fo and Fc are the observed and calculated structure factors, respectively.

[c] $R_{free} = \Sigma||Fo|-|Fc|| / \Sigma|Fo$ for 7% of the data not used at any stage of the structural refinement.

*Highest resolution shell is shown in parentheses.
