# Supplementary material for: Evolution of Minimal Specificity and Promiscuity in Steroid Hormone Receptors
Source: PLoS Genet. 2012 Nov 15;8(11):e1003072. doi: 10.1371/journal.pgen.1003072 (PMC3499368; doi:10.1371/journal.pgen.1003072)
Supplement: Table S7 — List of receptors and the organisms they were isolated from used in this study. (PDF) [file pgen.1003072.s020.pdf]

Table S7. List of receptors and the organisms they were isolated from used in the phylogenetic analyses. Receptors shaded in grey are those that were additionally incorporated into the phylogeny used to reconstruct AncSR1. ERs, estrogen receptors; PRs, progesterone receptors; ARs, androgen receptors; MRs, mineralocorticoid receptors; GRs, glucocorticoid receptors; ERRs, estrogen-related receptors; SF1, steroidogenic factor 1 receptor; RXR, retinoid X receptors; COUP-TFs, chicken ovalbumin upstream promoter transcription factors.

Species Name	Common Name	Phylum	Receptor Type	Abbreviation	Accession #	Database
Acanthopagrus schlegeli	black porgy	Vertebrata, Teleostei	AR	AcaSchAR	Q800S7	UniPROTKB/TrEMBL
			ERα	AcaSchERa	Q804Q6	UniPROTKB/TrEMBL
			ERβ	AcaSchERb	Q804Q7	UniPROTKB/TrEMBL
Alligator mississippiensis	American alligator	Vertebrate, Crocodylidae	ERα	AllMisERa	Q765N7	UniPROTKB/TrEMBL
			PR	AllMisPR	Q765N5	UniPROTKB/TrEMBL
Anguilla japonica	Japanese eel	Vertebrata, Teleostei	ARa	AngJapARa	Q9YGV9	UniPROTKB/TrEMBL
			ARb	AngJapARb	Q9PWG5	UniPROTKB/TrEMBL
			ERα	AngJapERa	O13012	UniPROTKB/TrEMBL
			ERβ	AngJapERb	O13012	UniPROTKB/TrEMBL
Aplysia californica	sea slug	Mollusca, Gastropoda	ER	AplCalER	Q6VU64	UniPROTKB/TrEMBL
Bos taurus	cow	Vertebrata, Certartiodactyla	ERα	BosTauERa	P49884	UniPROTKB/TrEMBL
			ERβ	BosTauERb	Q9XSB5	UniPROTKB/TrEMBL
			GR	BosTauGR	Q865Y6	UniPROTKB/TrEMBL
Branchiostoma floridae	amphioxus	Vertebrata, Cephalochordata	ER	BraFloER	B5L586	UniPROTKB/TrEMBL
			ERR	BraFloERR	Q5XTR0	UniPROTKB/TrEMBL
			SR	BraFloSR	B5SVV7	UniPROTKB/TrEMBL
Capitella capitata	bristle worm	Annelida, Polychaeta	ER	CapCapER	C0IRV2	UniPROTKB/TrEMBL
Candidia barbatus	Taiwanese cyprinid fish	Vertebrata, Teleostei	ERα	CanBarERa	Q7T3U5	UniPROTKB/TrEMBL
Canis familiaris	dog	Vertebrata, Carnivora	AR	CanFamAR	Q9TT90	UniPROTKB/TrEMBL
			ERα	CanFamERa	AAS20260	Genbank
			GR	CanFamGR	Q3HTT7	UniPROTKB/TrEMBL
			MR	CanFamMR	Q3HTT9	UniPROTKB/TrEMBL
			PR	CanFamPR	Q9GLW0	UniPROTKB/TrEMBL
Carassius auratus	goldfish	Vertebrata, Teleostei	AR	CarAurAR	Q8QFV2	UniPROTKB/TrEMBL
			ERα	CarAurERa	Q90WV1	UniPROTKB/TrEMBL
			ERβ1	CarAurERb1	Q9W669	UniPROTKB/TrEMBL
			ERβ2	CarAurERb2	Q9IAL9	UniPROTKB/TrEMBL
Cavia porcellus	guinea pig	Vertebrata, Euarchontoglires	ERα	CavPorERa	Q8CGK8	UniPROTKB/TrEMBL
			ERβ	CavPorERb	Q8CGK9	UniPROTKB/TrEMBL
			GR	CavPorGR	P49115	UniPROTKB/TrEMBL
Clarias gariepinus	African catfish	Vertebrata, Teleostei	ERα	CalGarERa	Q90WH6	UniPROTKB/TrEMBL
Cnemidophorus uniparens	whip-tailed lizard	Vertebrata, Squamata	ERα	CneUniERa	Q8UWA9	UniPROTKB/TrEMBL
			PR	CneUniPR	Q91425	UniPROTKB/TrEMBL
Conger myriaster	conger eel	Vertebrata, Teleostei	ERβ	ConMyrERb	Q762D6	UniPROTKB/TrEMBL
Coturnix coturnix	common quail	Vertebrata, Aves	ERα	CotCotERa	Q8AYH0	UniPROTKB/TrEMBL
			ERβ	CotCotERb	O93511	UniPROTKB/TrEMBL
Coturnix japonica	Asian quail	Vertebrata, Aves	AR	CotJapAR	Q689Y8	UniPROTKB/TrEMBL
Crassostrea gigas	Pacific oyster	Mollusca, Bivalvia	ER	CraGigER	A2A113	UniPROTKB/TrEMBL
Crocuta crocuta	hyena	Vertebrata, Carnivora	AR	CroCroAR	Q8MIK0	UniPROTKB/TrEMBL
Cyprinus carpio	common carp	Vertebrata, Teleostei	ERβ	CypCarERb	Q8JJB9	UniPROTKB/TrEMBL
Danio rerio	zebrafish	Vertebrata, Teleostei	AR	DanRerAR	Q6EAL5	UniPROTKB/TrEMBL
			COUP-TFII	DanRerCP2	O42532	UniPROTKB/TrEMBL
			ERα	DanRerERa	P57717	UniPROTKB/TrEMBL
			ERα2	DanRerER2a	Q7ZU32	UniPROTKB/TrEMBL
			ERβ1	DanRerERb1	Q90WS9	UniPROTKB/TrEMBL
			ERRα	DanRerERRa	Q6Q6F6	UniPROTKB/TrEMBL
			ERRβ	DanRerERRb	Q6Q6F5	UniPROTKB/TrEMBL
			ERRγ	DanRerERRg	Q6Q6F4	UniPROTKB/TrEMBL
			GR	DanRerGR	Q1XHK0	UniPROTKB/TrEMBL
			RXRα	DanRerRXRa	Q90416	UniPROTKB/TrEMBL
			RXRβ	DanRerRXRb	Q90417	UniPROTKB/TrEMBL
			RXRγ	DanRerRXRg	Q6DHP9	UniPROTKB/TrEMBL
Dicentrarchus labrax	European sea bass	Vertebrata, Teleostei	AR	DicLabAR	Q4G497	UniPROTKB/TrEMBL
			ERα	DicLabERa	Q6A4C2	UniPROTKB/TrEMBL
			ERβ	DicLabERb	Q6KF57	UniPROTKB/TrEMBL
Engystomops pustulosus	Lepodactylid frog	Vertebrata, Anura	AR	EngPusAR	Q2PK04	UniPROTKB/TrEMBL
Equus caballus	horse	Vertebrata, Perissodactyla	ERα	EquCabERa	Q9TV98	UniPROTKB/TrEMBL
Eublepharis macularius	leopard gecko	Vertebrata, Squamata	ERα	EubMacERa	Q2HWK5	UniPROTKB/TrEMBL
			ERβ	EubMacERb	Q2HWK4	UniPROTKB/TrEMBL
Felis catus	cat	Vertebrata, Carnivora	ERα	FelCatERa	Q53AD2	UniPROTKB/TrEMBL
Fundulus heteroclitus	killifish	Vertebrata, Teleostei	ERα	FunHetERa	Q7SZI0	UniPROTKB/TrEMBL
Gallus gallus	chicken	Vertebrata, Aves	COUP-TFII	GalGalCP2	Q90733	UniPROTKB/TrEMBL
			ERa	GalGalERa	P06212	UniPROTKB/TrEMBL
			ERβ	GalGalERb	Q9PTU5	UniPROTKB/TrEMBL
			ERRg	GalGalERRg	Q5UKY7	UniPROTKB/TrEMBL
			GR	GalGalGR	Q38HX7	UniPROTKB/TrEMBL
			MR	GalGalMR	C1KB84	UniPROTKB/TrEMBL
			PR	GalGalPR	P07812	UniPROTKB/TrEMBL
			SF1	GalGalSF1	Q9PW17	UniPROTKB/TrEMBL
Gambusia affinis	western mosquitofish	Vertebrata, Teleostei	AR	GamAffAR	Q60I32	UniPROTKB/TrEMBL
			ARa	GamAffARa	Q60GT9	UniPROTKB/TrEMBL
Gasterosteus aculeatus	three-spined stickelback	Vertebrata, Teleostei	ARb	GasAcuARb	Q801Z2	UniPROTKB/TrEMBL
Halichoeres tenuispinis	wrasse	Vertebrata, Teleostei	ERα	HalTenERa	Q7T2K8	UniPROTKB/TrEMBL
			ERβ	HalTenERb	Q7T2K7	UniPROTKB/TrEMBL
Halichoeres trimaculatus	protogynous wrasse	Vertebrata, Teleostei	AR	HalTriAR	Q9DDJ4	UniPROTKB/TrEMBL
			ERα	HalTriERa	Q9DDJ3	UniPROTKB/TrEMBL
Haplochromis burtoni	African cichlid	Vertebrata, Teleostei	AR	HapBurAR	Q9W6F4	UniPROTKB/TrEMBL
			ARb	HapBurARb	Q8QFV7	UniPROTKB/TrEMBL
			ERα	HapBurERa	Q6TGB3	UniPROTKB/TrEMBL
			GR1	HapBur1GR	Q8JJ92	UniPROTKB/TrEMBL
			GR2	HapBur2aGR	Q8JJ91	UniPROTKB/TrEMBL
			MR	HapBurMR	Q8JJ89	UniPROTKB/TrEMBL
Homo sapiens	human	Vertebrata, Primates	AR	HomSapAR	P10275	UniPROTKB/TrEMBL
			COUP-TF1	HomSapCP1	P10589	UniPROTKB/TrEMBL
			COUP-TFII	HomSapCP2	P24468	UniPROTKB/TrEMBL
			ERα	HomSapERa	P03372	UniPROTKB/TrEMBL
			ERβ	HomSapERb	Q9UHD3	UniPROTKB/TrEMBL
			ERRα	HomSapERR1	P11474	UniPROTKB/TrEMBL
			ERRβ	HomSapERR2	O95718	UniPROTKB/TrEMBL
			ERRγ	HomSapERR3	P62508	UniPROTKB/TrEMBL
			GR	HomSapGR	P04150	UniPROTKB/TrEMBL
			MR	HomSapMR	P08235	UniPROTKB/TrEMBL
			PR	HomSapPR	P06401	UniPROTKB/TrEMBL
			RXRα	HomSapRXRa	P19793	UniPROTKB/TrEMBL
			RXRβ	HomSapRXRb	P28702	UniPROTKB/TrEMBL
			RXRγ	HomSapRXRg	P48443	UniPROTKB/TrEMBL
			SF1	HomSapSF1	Q13285	UniPROTKB/TrEMBL
Ictalurus punctatus	channel catfish	Vertebrata, Teleostei	ERa	IctPunERa	Q9YHZ7	UniPROTKB/TrEMBL
			ERβ	IctPunERb	Q9IAK1	UniPROTKB/TrEMBL
Kryptolebias marmoratus	mangrove rivulus	Vertebrata, Teleostei	AR	KryMarAR	Q15HT9	UniPROTKB/TrEMBL
			ERα	KryMarERa	Q0KKB8	UniPROTKB/TrEMBL
			ERβ	KryMarERb	Q0KKB9	UniPROTKB/TrEMBL
Leucoraja erinacea	little skate	Vertebrata, Elasmobranchii	AR	LeuEriAR	Q1KXY2	UniPROTKB/TrEMBL
			GR	LeuEriGR	Q1KXY4	UniPROTKB/TrEMBL
			MR	LeuEriMR	Q1KXY3	UniPROTKB/TrEMBL
			PR	LeuEriPR	Q1KXY1	UniPROTKB/TrEMBL
Lottia gigantea	limpet	Mollusca, Gastropoda	ER	LotGigER	Lotgi1:132166	JGI
Manacus vitellinus	gold-collared manakin	Vertebrata, Aves	AR	ManVitAR	Q4U102	UniPROTKB/TrEMBL
Marisa cornuarietis	apple snail	Mollusca, Gastropoda	ER	MarCorER	Q06AL2	UniPROTKB/TrEMBL
Mesocricetus auratus	golden hamster	Vertebrata, Rodentia	ERα	MesAurERa	Q9QZJ5	UniPROTKB/TrEMBL
Microtus ochrogaster	priarie vole	Vertebrata, Rodentia	ERα	MicOchERa	Q1EDB6	UniPROTKB/TrEMBL
Micropterus salmoides	largemouth bass	Vertebrata, Teleostei	ERα	MicSalERa	Q9DDZ4	UniPROTKB/TrEMBL
			ERβ	MicSalERb	Q6XSH2	UniPROTKB/TrEMBL
Micropogonias undulatus	Atlantic croaker	Vertebrata, Teleostei	AR	MicUndAR	Q66VR6	UniPROTKB/TrEMBL
			ERβ	MicUndERb	P57781	UniPROTKB/TrEMBL
			ERγ	MicUndERg	P57783	UniPROTKB/TrEMBL
Mus musculus	mouse	Vertebrata, Rodentia	ERα	MusMusERa	P19785	UniPROTKB/TrEMBL
			ERβ	MusMusERb	O08537	UniPROTKB/TrEMBL
			ERRα	MusMusERR1	O08580	UniPROTKB/TrEMBL
			ERRβ	MusMusERR2	Q61539	UniPROTKB/TrEMBL
			ERRg	MusMusERR3	P62509	UniPROTKB/TrEMBL
			GR	MusMusGR	P06537	UniPROTKB/TrEMBL
			MR	MusMusMR	Q8VII8	UniPROTKB/TrEMBL
			PR	MusMusPR	Q00175	UniPROTKB/TrEMBL
			SF1	MusMusSF1	P33242	UniPROTKB/TrEMBL
Myxine glutinosa	Atlantic hagfish	Vertebrata, Teleostei	CR	MyxGluCR	Q1KXY6	UniPROTKB/TrEMBL
			ER	MyxGluER	EU439936	Genbank
			SR	MyxGluSR	Q1KXY5	UniPROTKB/TrEMBL
Nucella lapillus	Atlantic dogwinkle	Mollusca, Gastropoda	ER	NucLapER	A5YVJ5	UniPROTKB/TrEMBL
Octopus vulgaris	octopus	Mollusca, Cephalopoda	ER	OctVulER	Q19AB0	UniPROTKB/TrEMBL
Oncorhynchus mykiss	rainbow trout	Vertebrata, Teleostei	AR	OncMykARa2	O93244	UniPROTKB/TrEMBL
			AR	OncMykARb2	O93245	UniPROTKB/TrEMBL
			ERα	OncMykERa	P16058	UniPROTKB/TrEMBL
			ERβ	OncMykERb	P57782	UniPROTKB/TrEMBL
			GR	OncMykGR	P49843	UniPROTKB/TrEMBL
			GR	OncMykGR2	Q6RKQ3	UniPROTKB/TrEMBL
			MR	OncMykMR	Q5WP02	UniPROTKB/TrEMBL
Onychostoma barbatula	cyprinid fish	Vertebrata, Teleostei	ERβ	OnyBarERb2	Q2PDJ6	UniPROTKB/TrEMBL
Oreochromis aureus	Israel tilapia	Vertebrata, Teleostei	ERα	OreAurERa	P50240	UniPROTKB/TrEMBL
Orechromis niloticus	Nile tilapia	Vertebrata, Teleostei	AR	OreNilARa	Q8UWB8	UniPROTKB/TrEMBL
			AR	OreNilARb	Q8UWB7	UniPROTKB/TrEMBL
			ERα	OreNilERa	Q9YH33	UniPROTKB/TrEMBL
			ERβ	OreNilERb	Q7T3U4	UniPROTKB/TrEMBL
Oryctolagus cuniculus	rabbit	Vertebrata, Glires	AR	OrtCunAR	P49699	UniPROTKB/TrEMBL
			GR	OrtCunGR	P59667	UniPROTKB/TrEMBL
			PR	OrtCunPR	P06186	UniPROTKB/TrEMBL
Oryzias latipes	Japanese ricefish	Vertebrata, Teleostei	AR	OryLatAR	Q76LM5	UniPROTKB/TrEMBL
			ERα	OryLatERa	P50241	UniPROTKB/TrEMBL
			ERβ	OryLatERb	Q8UW75	UniPROTKB/TrEMBL
Ovis aries	sheep	Vertebrata, Ruminantia	ERα	OviAriERa	Q95L13	UniPROTKB/TrEMBL
			ERβ	OviAriERb	Q9TU15	UniPROTKB/TrEMBL
Pagrus major	red sea bream	Vertebrata, Teleostei	AR	PagMajAR	O93497	UniPROTKB/TrEMBL
			ERα	PagMajERa	O42132	UniPROTKB/TrEMBL
Paralichthys olivaceus	Japanses flounder	Vertebrata, Teleostei	ERα	ParOliERa	Q8QHL0	UniPROTKB/TrEMBL
			ERβ	ParOliERb	Q8QHK9	UniPROTKB/TrEMBL
			GR	ParOliGR	O73673	UniPROTKB/TrEMBL
Petromyzon marinus	sea lamprey	Vertebrata, Hyperoartia	COUP-TFII	PetMarCP2	Q42509	UniPROTKB/TrEMBL
			CR	PetMarCR	Q90ZM7	UniPROTKB/TrEMBL
			ER	PetMarER	Q90ZM8	UniPROTKB/TrEMBL
			PR	PetMarPR	Q90ZM6	UniPROTKB/TrEMBL
			RXR2	PetMarRXR2	Q90Y01	UniPROTKB/TrEMBL
Pimephales promelas	fathead minnow	Vertebrata, Teleostei	AR	PimProAR	Q9I8F5	UniPROTKB/TrEMBL
			ERα	PimProERa	Q5XXP1	UniPROTKB/TrEMBL
			GR	PimProGR	Q2TU59	UniPROTKB/TrEMBL
Platynereis dumerilli	polychaete worm	Annelida, Polychaeta	ER	PlaDumER	EU482033	Genbank
Rana catesbeiana	bullfrog	Vertebrata, Anura	AR	RanCatAR	Q7T1K4	UniPROTKB/TrEMBL
Rana dybowskii	Korean brown frog	Vertebrata, Anura	PR	RanDybPR	Q8AYI2	UniPROTKB/TrEMBL
Rana rugosa	wrinkled frog	Vertebrata, Anura	SF1	RanRugSF1	Q9YI95	UniPROTKB/TrEMBL
Rattus norvegicus	rat	Vertebrata, Rodentia	AR	RatNovAR	P15207	UniPROTKB/TrEMBL
			ERβ	RatNovERb	Q62986	UniPROTKB/TrEMBL
			ERRβ	RatNovERR2	P11475	UniPROTKB/TrEMBL
			GR	RatNovGR	P06536	UniPROTKB/TrEMBL
			MR	RatNovMR	P22199	UniPROTKB/TrEMBL
			PR	RatNovPR	Q63449	UniPROTKB/TrEMBL
Saimiri boliviensis	squirrel monkey	Vertebrata, Primates	GR	SaiBolGR	O13186	UniPROTKB/TrEMBL
			MR	SaiBolMR	Q4JM28	UniPROTKB/TrEMBL
Salmo salar	Atlantic salmon	Vertebrata, Teleostei	ERβ	SalSalERb	Q6R7S4	UniPROTKB/TrEMBL
Salmo trutta	brown salmon	Vertebrata, Teleostei	GR	SalTruGR	Q5I1Z4	UniPROTKB/TrEMBL
Serinus canarius	canary	Vertebrata, Aves	AR	SerCanAR	Q91445	UniPROTKB/TrEMBL
Sparus aurata	gilthead seabream	Vertebrata, Teleostei	ERα	SpaAurERa	Q9PVZ9	UniPROTKB/TrEMBL
			ERβ	SpaAurERb	Q9W6M2	UniPROTKB/TrEMBL
			GR	SpaAurGR	Q1HGK6	UniPROTKB/TrEMBL
Spinibarbus denticulatus	cyprinid fish	Vertebrata, Teleostei	ERβ	SpiDebERb	Q1HCL3	UniPROTKB/TrEMBL
Squalus acanthias	spiny dogfish	'ertebrata, Elasmobranch	AR	SquAcaAR	Q56VU2	UniPROTKB/TrEMBL
			ERβ	SquAcaERb	Q90ZE6	UniPROTKB/TrEMBL
Sturnus vulgaris	European starling	Vertebrata, Aves	ERβ	StuVulERb	Q9PVE2	UniPROTKB/TrEMBL
Sus scrofa	pig	'ertebrata, Certartiodactyl	AR	SusScrAR	Q9GKL7	UniPROTKB/TrEMBL
			ERα	SusScrERa	Q29040	UniPROTKB/TrEMBL
			ERβ	SusScrERb	Q9XSW2	UniPROTKB/TrEMBL
			GR	SusScrGR	Q9N1U3	UniPROTKB/TrEMBL
Taeniopygia guttata	zebrafinch	Vertebrata, Aves	AR	TaeGutAR	Q2VP86	UniPROTKB/TrEMBL
			ERα	TaeGutERa	Q91250	UniPROTKB/TrEMBL
			MR	TaeGutMR	Q157N1	UniPROTKB/TrEMBL
			SF1	TaeGutSF1	Q90XC4	UniPROTKB/TrEMBL
Takifugu rubripes	pufferfish	Vertebrata, Teleostei	AR	TakRubAR	NEWSINFRUP00000156318	Ensemble
Tetraodon nigroviridis	green-spotted pufferfish	Vertebrata, Teleostei	ERα	TetNigERa	Q4S6Y6	UniPROTKB/TrEMBL
Thais clavigera	whelk	Mollusca, Gastropoda	ER	ThaClaER	Q86B27	UniPROTKB/TrEMBL
Trichoplax adhaerens		Placozoa	ERR	TriAdhERR	Triad1:16711	JGI
Tupaia glis	common tree shrew	Vertebrata, Scandentia	GR	TupGliGR	Q95267	UniPROTKB/TrEMBL
			MR	TupGliMR	Q29131	UniPROTKB/TrEMBL
Xenopus laevis	African clawed toad	Vertebrata, Anura	AR	XenLaeAR	P70048	UniPROTKB/TrEMBL
			CP1	XenLaeCP1	Q9W745	UniPROTKB/TrEMBL
			ERα	XenLaeERa	P81559	UniPROTKB/TrEMBL
			ERR	XenLaeERR	Q6AX97	UniPROTKB/TrEMBL
			GR	XenLaeGR	P49844	UniPROTKB/TrEMBL
			MR	XenLaeMR	Q66J29	UniPROTKB/TrEMBL
			PR	XenLaePR	Q9DDU9	UniPROTKB/TrEMBL
			RXRα	XenLaeRXRa	P51128	UniPROTKB/TrEMBL
			RXRβ	XenLaeRXRb	Q91840	UniPROTKB/TrEMBL
			RXRγ	XenLaeRXRg	P51129	UniPROTKB/TrEMBL
Xenopus tropicalis	clawed toad	Vertebrata, Anura	ERα	XenTroERa	Q6W5G9	UniPROTKB/TrEMBL
			ERβ	XenTroERb	Q6W5G8	UniPROTKB/TrEMBL
Zoarces viviparus	Eelpout	Vertebrata, Teleostei	ERα	ZoaVivERa	Q800Q2	UniPROTKB/TrEMBL
