# Supplementary material for: Evolution of Minimal Specificity and Promiscuity in Steroid Hormone Receptors
Source: PLoS Genet. 2012 Nov 15;8(11):e1003072. doi: 10.1371/journal.pgen.1003072 (PMC3499368; doi:10.1371/journal.pgen.1003072)
Supplement: Table S8 — Comparison of the sequence of AncSR2 as reconstructed on the ML phylogeny and gene duplication phylogeny. (PDF) [file pgen.1003072.s021.pdf]

Table S8. Comparison of the sequence of AncSR2 as reconstructed on the ML phylogeny and gene duplication phylogeny (see Figs. S10, S12). Residues in the ligand binding pocket are indicated in red text. Differences between the two ancestral reconstructions are highlighted in orange.

	AncSR2 as reconstructed on 184-taxa ML tree				AncSR2 as reconstructed on 184-taxa gene duplication tree			
	Amino acid	PP1	Alt. State	PP2	Amino acid	PP1	Alt. State	PP2
1	P	1.00			P	1.00		
2	S	0.81	T	0.19	S	0.64	T	0.34
3	L	0.91	I	0.04	L	0.98	I	0.01
4	I	0.78	V	0.21	I	0.81	V	0.19
5	S	0.94	T	0.05	S	0.98	T	0.01
6	I	0.94	V	0.03	I	0.99	V	0.01
7	L	1.00			L	1.00		
8	Q	0.50	E	0.49	Q	0.99	E	0.01
9	A	0.56	V	0.19	A	0.54	V	0.21
10	I	1.00			I	1.00		
11	E	1.00			E	1.00		
12	P	1.00			P	1.00		
13	E	0.99	D	0.01	E	1.00	D	0.01
14	V	0.96	I	0.04	V	0.98	I	0.02
15	V	0.98	I	0.02	V	0.99	I	0.01
16	Y	1.00	F	0.00	Y	1.00	F	0.00
17	A	1.00			A	1.00		
18	G	1.00			G	1.00		
19	Y	1.00	F	0.00	Y	0.99	F	0.01
20	D	1.00			D	1.00		
21	N	0.81	S	0.19	N	1.00	S	0.00
22	T	0.61	S	0.38	T	0.71	S	0.29
23	Q	0.97	R	0.02	Q	0.96	R	0.03
24	P	1.00			P	1.00		
25	D	0.95	E	0.04	D	0.90	E	0.08
26	T	1.00			T	1.00		
27	T	0.99	S	0.01	T	0.99	S	0.01
28	N	0.97	S	0.02	N	0.96	S	0.03
29	Y	0.80	H	0.19	Y	0.99	H	0.01
30	L	1.00	M	0.00	L	1.00	M	0.00
31	L	1.00			L	1.00		
32	S	0.90	T	0.10	S	0.86	T	0.14
33	S	1.00	T	0.00	S	1.00		
34	L	1.00			L	1.00		
35	N	1.00			N	1.00		
36	R	0.99	K	0.01	R	1.00	K	0.00
37	L	1.00			L	1.00		
38	A	0.87	C	0.06	C	1.00	A	0.00
39	E	0.85	G	0.13	E	0.99	D	0.01
40	K	0.61	R	0.40	K	0.62	R	0.38
41	Q	1.00			Q	1.00		
42	L	0.77	M	0.23	L	1.00	M	0.00
43	V	1.00	I	0.00	V	1.00		
44	S	0.92	R	0.05	S	0.94	R	0.03
45	V	1.00	I	0.00	V	0.99	I	0.01
46	V	1.00			V	1.00		
47	K	1.00	R	0.00	K	1.00		
48	W	1.00			W	1.00		
49	A	1.00			A	1.00		
50	K	1.00			K	1.00		
51	A	0.88	V	0.08	A	0.90	S	0.06
52	L	1.00	M	0.00	L	1.00	M	0.00
53	P	1.00			P	1.00		
54	G	1.00			G	1.00		
55	F	1.00			F	1.00		
56	R	1.00	K	0.00	R	1.00	K	0.00
57	N	0.99	S	0.01	N	1.00	S	0.00
58	L	1.00			L	1.00		
59	H	0.98	P	0.01	H	1.00		
60	L	0.79	I	0.21	I	1.00	L	0.00
61	D	0.99	E	0.01	D	1.00		
62	D	1.00			D	1.00		
63	Q	1.00			Q	1.00		
64	M	1.00			M	1.00		
65	T	1.00			T	1.00		
66	L	1.00			L	1.00		
67	I	0.52	L	0.48	I	1.00		
68	Q	1.00			Q	1.00		
69	Y	1.00			Y	1.00		
70	S	1.00			S	1.00		
71	W	1.00			W	1.00		
72	M	1.00			M	1.00		
73	G	0.75	S	0.14	G	1.00		
74	L	1.00			L	1.00		
75	M	1.00			M	1.00		
76	A	0.88	S	0.05	A	0.94	V	0.03
77	F	1.00			F	1.00		
78	A	0.84	S	0.15	A	1.00	S	0.00
79	L	0.59	M	0.41	M	1.00	L	0.00
80	G	0.92	S	0.07	G	0.99	S	0.01
81	W	1.00			W	1.00		
82	R	1.00			R	1.00		
83	S	1.00			S	1.00		

	AncSR2 as reconstructed on 184-taxa ML tree				AncSR2 as reconstructed on 184-taxa gene duplication tree			
	Amino acid	PP	Alt. State	PP	Amino acid	PP	Alt. State	PP
84	Y	1.00			Y	1.00		
85	K	0.99	R	0.01	K	0.99	R	0.00
86	H	1.00			H	1.00		
87	T	0.82	A	0.07	T	0.86	V	0.07
88	N	1.00			N	1.00		
89	G	1.00	S	0.00	G	1.00	S	0.00
90	Q	0.76	K	0.13	Q	0.80	K	0.18
91	M	1.00	L	0.00	M	1.00	L	0.00
92	L	1.00			L	1.00		
93	Y	1.00	F	0.00	Y	1.00		
94	F	1.00			F	1.00		
95	A	1.00			A	1.00		
96	P	1.00			P	1.00		
97	D	1.00			D	1.00		
98	L	1.00			L	1.00		
99	I	0.98	V	0.02	I	0.99	V	0.01
100	F	1.00			F	1.00		
101	N	1.00			N	1.00		
102	E	1.00			E	1.00		
103	Q	0.63	E	0.36	Q	0.98	E	0.02
104	R	1.00			R	1.00		
105	M	1.00			M	1.00		
106	Q	0.99	H	0.00	Q	1.00	K	0.00
107	Q	1.00			Q	1.00	K	0.00
108	S	1.00			S	1.00		
109	A	1.00	T	0.00	A	1.00		
110	M	1.00			M	1.00		
111	Y	1.00	F	0.00	Y	1.00		
112	D	0.73	E	0.27	D	0.75	E	0.25
113	L	1.00			L	1.00		
114	C	1.00			C	1.00		
115	Q	0.48	L	0.18	L	0.46	M	0.22
116	G	1.00			G	1.00		
117	M	1.00			M	1.00		
118	Q	0.48	R	0.48	R	0.77	Q	0.22
119	Q	0.99	K	0.01	Q	1.00	N	0.00
120	I	1.00	V	0.00	I	1.00	V	0.01
121	S	0.99	A	0.01	S	1.00		
122	Q	0.47	L	0.11	Q	0.68	E	0.17
123	E	1.00	D	0.00	E	1.00		
124	F	1.00			F	1.00		
125	V	0.87	I	0.07	V	0.81	I	0.10
126	R	0.98	K	0.02	R	0.98	K	0.02
127	L	1.00			L	1.00	M	0.00
128	Q	1.00			Q	1.00		
129	V	0.97	L	0.03	V	1.00	I	0.00
130	T	0.98	S	0.02	T	0.98	S	0.02
131	Q	0.53	H	0.28	Q	0.85	H	0.10
132	E	1.00	D	0.01	E	0.99	D	0.01
133	E	1.00			E	1.00		
134	F	0.99	Y	0.01	F	1.00		
135	L	1.00			L	1.00		
136	C	1.00			C	1.00		
137	M	1.00			M	1.00		
138	K	1.00			K	1.00		
139	A	0.87	V	0.13	A	1.00		
140	L	0.98	I	0.02	L	0.96	I	0.04
141	L	1.00			L	1.00		
142	L	1.00			L	1.00		
143	L	1.00			L	1.00		
144	S	0.99	N	0.01	S	0.98	N	0.02
145	T	1.00			T	1.00		
146	V	0.67	I	0.33	V	0.59	I	0.41
147	P	1.00			P	1.00		
148	K	0.61	Q	0.34	Q	0.72	K	0.25
149	E	0.54	D	0.46	E	0.98	D	0.02
150	G	1.00			G	1.00		
151	L	1.00			L	1.00		
152	K	1.00	R	0.00	K	1.00		
153	S	1.00	T	0.00	S	1.00	T	0.00
154	Q	1.00	H	0.00	Q	1.00		
155	A	0.83	T	0.13	A	0.61	T	0.31
156	S	0.34	Y	0.17	S	0.33	Y	0.32
157	F	1.00			F	1.00		
158	D	0.95	E	0.05	D	0.93	E	0.07
159	E	1.00	D	0.00	E	1.00	D	0.00
160	M	0.99	I	0.01	M	1.00		
161	R	1.00			R	1.00		
162	M	1.00	I	0.00	M	1.00	I	0.00
163	N	0.98	S	0.02	N	0.98	S	0.02
164	Y	1.00			Y	1.00		
165	I	1.00			I	1.00		
166	K	0.68	R	0.32	R	0.98	K	0.02

	AncSR2 as reconstructed on 184-taxa ML tree				AncSR2 as reconstructed on 184-taxa gene duplication tree			
	Amino acid	PP	Alt. State	PP	Amino acid	PP	Alt. State	PP
167	E	1.00			E	1.00		
168	L	1.00			L	1.00		
169	N	0.43	R	0.25	N	0.95	S	0.03
170	R	0.66	K	0.34	R	0.80	K	0.20
171	A	0.68	V	0.31	A	0.75	V	0.24
172	I	0.94	V	0.06	I	0.94	V	0.06
173	A	0.68	V	0.20	A	0.73	V	0.11
174	K	0.55	R	0.34	R	0.47	K	0.32
175	K	0.55	R	0.24	K	0.51	Q	0.24
176	E	0.98	D	0.02	E	0.97	D	0.03
177	N	0.85	K	0.08	N	0.78	K	0.19
178	N	1.00	S	0.00	N	1.00	S	0.00
179	S	0.53	T	0.24	A	0.34	S	0.28
180	A	0.47	G	0.24	A	0.74	V	0.16
181	Q	0.99	E	0.01	Q	0.98	E	0.02
182	N	0.56	S	0.41	S	0.80	N	0.15
183	W	1.00			W	1.00		
184	Q	1.00	H	0.00	Q	1.00		
185	R	1.00			R	1.00		
186	F	1.00			F	1.00		
187	Y	1.00			Y	1.00		
188	Q	1.00			Q	1.00		
189	L	1.00			L	1.00		
190	T	1.00			T	1.00		
191	K	1.00			K	1.00		
192	L	0.99	M	0.01	L	1.00	M	0.00
193	L	1.00			L	1.00		
194	D	1.00			D	1.00		
195	S	1.00			S	1.00		
196	M	1.00			M	1.00		
197	H	1.00			H	1.00		
198	D	0.97	E	0.03	D	0.99	E	0.01
199	L	1.00	M	0.00	L	1.00		
200	V	1.00			V	1.00		
201	G	1.00	E	0.00	G	1.00	S	0.00
202	G	0.93	K	0.05	G	0.87	K	0.12
203	L	1.00			L	1.00		
204	L	1.00			L	1.00		
205	Q	0.99	E	0.01	Q	0.97	E	0.03
206	F	1.00			F	1.00		
207	C	1.00			C	1.00		
208	F	1.00			F	1.00		
209	Y	0.60	H	0.30	H	0.60	N	0.22
210	T	1.00			T	1.00		
211	F	1.00			F	1.00		
212	V	0.99	I	0.01	V	0.99	I	0.00
213	Q	0.90	E	0.10	Q	1.00		
214	S	1.00	N	0.00	S	1.00		
215	Q	0.82	K	0.17	Q	1.00	K	0.00
216	A	0.95	T	0.03	A	0.96	T	0.03
217	L	1.00	M	0.00	L	1.00	W	0.00
218	S	0.96	K	0.01	S	1.00	A	0.00
219	V	1.00			V	1.00		
220	E	1.00			E	1.00		
221	F	1.00			F	1.00		
222	P	1.00			P	1.00		
223	E	1.00	D	0.00	E	1.00	D	0.00
224	M	1.00			M	1.00		
225	L	0.77	M	0.23	M	1.00	L	0.00
226	V	0.63	S	0.18	S	0.70	A	0.26
227	E	1.00			E	1.00		
228	I	1.00	M	0.00	I	1.00		
229	I	1.00			I	1.00		
230	S	1.00	N	0.00	S	1.00	T	0.00
231	A	0.90	D	0.07	A	1.00		
232	Q	1.00			Q	1.00		
233	L	1.00	M	0.00	L	1.00	M	0.00
234	P	1.00			P	1.00		
235	K	0.95	R	0.05	K	0.89	R	0.11
236	V	0.86	I	0.14	V	0.75	I	0.25
237	L	0.49	M	0.22	L	0.97	M	0.02
238	A	0.92	S	0.05	A	0.98	S	0.01
239	G	1.00			G	1.00		
240	M	0.97	L	0.01	M	0.98	L	0.01
241	A	0.96	T	0.03	A	1.00	V	0.00
242	K	1.00	R	0.00	K	1.00	R	0.00
243	P	1.00	S	0.00	P	1.00	A	0.00
244	L	1.00			L	1.00		
245	L	0.99	F	0.00	L	0.99	H	0.01
246	F	1.00			F	1.00		
247	H	1.00			H	1.00		
248	K	0.91	R	0.07	K	0.97	R	0.03
249	K	1.00	R	0.00	K	1.00		
	Mean Posterior Probability	0.93			Mean Posterior Probability	0.97		
